# A platform for integrated spectrometers based on solution-processable semiconductors

**DOI:** 10.1038/s41377-023-01231-1

**Published:** 2023-07-26

**Authors:** Yanhao Li, Xiong Jiang, Yimu Chen, Yuhan Wang, Yunkai Wu, De Yu, Kaiyang Wang, Sai Bai, Shumin Xiao, Qinghai Song

**Affiliations:** 1 Ministry of Industry and Information Technology Key Lab of Micro-Nano Optoelectronic Information System, Guangdong Provincial Key Laboratory of Semiconductor Optoelectronic Materials and Intelligent Photonic Systems, Harbin Institute of Technology (Shenzhen), Shenzhen, Guangdong, 518055 China; 2 Institute of Fundamental and Frontier Sciences, University of Electronic Science and Technology of China, Chengdu, Sichuan 611731 China; 3 Collaborative Innovation Center of Extreme Optics, Shanxi University, Taiyuan, Shan`xi 030006 China

**Keywords:** Optoelectronic devices and components, Imaging and sensing, Photonic devices

## Abstract

Acquiring real-time spectral information in point-of-care diagnosis, internet-of-thing, and other lab-on-chip applications require spectrometers with hetero-integration capability and miniaturized feature. Compared to conventional semiconductors integrated by heteroepitaxy, solution-processable semiconductors provide a much-flexible integration platform due to their solution-processability, and, therefore, more suitable for the multi-material integrated system. However, solution-processable semiconductors are usually incompatible with the micro-fabrication processes. This work proposes a facile and universal platform to fabricate integrated spectrometers with semiconductor substitutability by unprecedently involving the conjugated mode of the bound states in the continuum (conjugated-BIC) photonics. Specifically, exploiting the conjugated-BIC photonics, which remains unexplored in conventional lasing studies, renders the broadband photodiodes with ultra-narrowband detection ability, detection wavelength tunability, and on-chip integration ability while ensuring the device performance. Spectrometers based on these ultra-narrowband photodiode arrays exhibit high spectral resolution and wide/tunable spectral bandwidth. The fabrication processes are compatible with solution-processable semiconductors photodiodes like perovskites and quantum dots, which can be potentially extended to conventional semiconductors. Signals from the spectrometers directly constitute the incident spectra without being computation-intensive, latency-sensitive, and error-intolerant. As an example, the integrated spectrometers based on perovskite photodiodes are capable of realizing narrowband/broadband light reconstruction and in-situ hyperspectral imaging.

## Introduction

The growing need of acquiring real-time spectral information in point-of-care medical diagnosis, internet-of-things, all-optical information processing, and other lab-on-chip applications have brought the requests of developing spectrometers with hetero-integration capability and miniaturized footprints^1–4^. Depending on the working mechanism, integrated spectrometers can be classified into wavelength multiplexing mode and wavelength de-multiplexing mode. Wavelength multiplexing spectrometers reconstruct spectra by post-processing with algorithms, which have been shown to possess facile device configuration, high spectral accuracy/resolution, and a small footprint^1,3–5^. Contrarily, wavelength de-multiplexing spectrometers directly constitute spectra by selectively detecting lights without additional data-processing chips^6,7^, which is favorable for the abovementioned applications that require a high level of integration and low power consumption. However, complicated micro-/nano-fabrication processes to achieve wavelength de-multiplexing integration and miniaturization are usually heavily demanded. Thus, current studies mainly focus on process-compatible conventional semiconductors like Si and Ge^6,8,9^. For lab-on-chip applications that are consisted of multiple types of functional sub-devices, hetero-integrating various epitaxial conventional semiconductors inevitably brings difficulties and high costs. Novel semiconductors, like perovskites, organics, quantum dots, and others, possess advantages in facile solution-processability and cost, as well as spectral tunability and functional diversity^10,11^. These properties are highly compatible with the requirement of hetero-integration, rendering them with great potential in next-generation integrated spectrometers. However, these materials are usually incompatible with the micro-/nano-fabrication processes, therefore, hindering their development as integrated spectrometers. Although the unique filterless narrowband mechanisms in the novel semiconductors can be applied to differentiate light with different wavelengths without abundant micro-/nano-fabrication processes, the low spectral resolution and tedious sequential deposition processes limit their actual application.

Herein, a novel platform to realize integrated spectrometers that applies to various semiconductor materials and are suitable for miniaturization and on-chip integration is demonstrated. In this platform, the concept of the conjugated mode of the bound states in the continuum (conjugated-BIC), which remains unexplored in the conventional BIC studies like lasing^12–16^, is employed to extract monochromatic lights with ultra-narrow linewidth and broad/tunable bandwidth. The extracted lights are then collected by the planar-integrated broadband semiconductor photodiodes for monochromatic signal output, which can directly constitute the incident spectra without further computational efforts.

Specifically, integrated spectrometers based on perovskite photodiode arrays are demonstrated as an example due to their outstanding properties^11,16–22^. Each perovskite photodiode and conjugated-BIC photonic device consist of an ultra-narrowband photodiode corresponding to a specific monochromatic wavelength detection. Owing to the proposed platform, the ultra-narrowband perovskite photodiodes exhibit a spectral resolution as narrow as 3.6 nm. Responsivity, specified detectivity, and response speed of the ultra-narrowband perovskite photodetectors reach 0.2 A W^−1^, 5.1 × 10^11^ Jones, and 100 ns, respectively. These performance are also superior to the state-of-the-art perovskite narrowband photodiodes^23–25^. Based on the ultra-narrowband photodiode arrays, integrated perovskite spectrometers show the capability of precisely reconstructing both broadband and narrowband spectra, as well as realizing hyperspectral imaging.

## Results

### Device design and working principle

The concept of conjugated-BIC is employed in this study, which is largely ignored in past BIC studies like lasing. In principles, BIC mode, first discovered in quantum mechanics, occurs due to the direct and via-the-continuum interaction between quasi-stationary states and possesses infinitely large Q factors and ultra-narrow resonant linewidth^12,13^. When two resonances approach one another in a photonic system, they can be described with a 2 × 2 non-Hermitian Hamiltonian $$H=\left(\begin{array}{cc}{E}_{1} & W\\ V & {E}_{2}\end{array}\right)$$ where ${E}_{1,2}$ and $\sqrt{{VW}}$ are the energy of two states and their coupling constant. The eigenvalues are thus expressed as ${E}_{\pm }=\frac{{E}_{1}+{E}_{2}}{2}\pm \sqrt{{\left(\frac{{E}_{1}-{E}_{2}}{2}\right)}^{2}+{VW}}$. In conventional studies, especially lasing experiments, researchers mostly focus on one state that has the highest Q factor (also known as BIC)^14–16,26^. However, the highest Q mode corresponds to decoupling to the external supercontinuum and is hard to be excited with external illumination, indicating that this mode is difficult to be used in photodetection. In this case, we, for the first time, point out the importance of the conjugated-BIC. Due to the constructive interference in the decay channel, this conjugated mode experiences higher leakage and has relatively low Q (the absolute linewidth is still ultra-narrow, theoretically can be far below 1 nm). Considering a time-reversal process, this conjugated mode can be easily excited, and the theoretical coupling efficiency is as high as 100%, showing the potential to be employed in narrowband photodetection and spectrometer. Consequently, only the conjugated-BIC mode with ultra-narrow linewidth can be coupled into the waveguide and be conducted to the perovskite regions for ultra-narrowband signal conversion, while all the others shall directly transmit through the grating without producing any signals. In conclusion, the conjugated-BIC photonic gratings are capable of extracting monochromatic lights, and their wavelength can be facilely tuned by adjusting the grating structure.

**Fig. 1:**
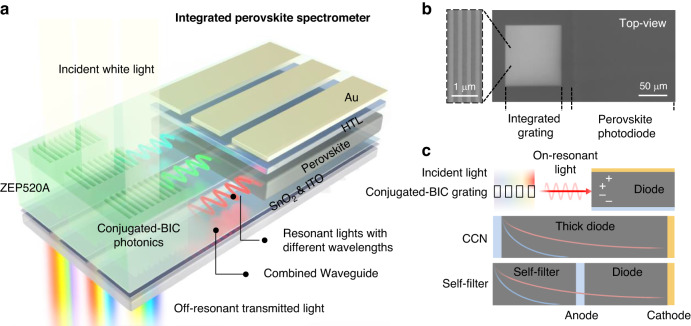
Design and working principles of the integrated spectrometers. **a** The schematic working mechanism of the integrated spectrometer. Conjugated-BIC photonics and combined waveguides are planarly integrated with perovskite photodiode arrays. Monochromatic light with a specific wavelength can be coupled into the waveguide and propagate to the corresponding photodiode for an ultra-narrowband response. **b** Top-view SEM image of an ultra-narrowband photodiode that constitutes the integrated spectrometer. Zoom-in SEM image: details of the grating. **c** Schematic mechanism comparison between our mechanism (upper panel) and the conventional mechanisms (middle and lower panels). Curves represent the photocarrier density generated by lights with different wavelengths

As schematically shown in Fig. 1a, the integrated spectrometer is composed of ultra-narrowband photodiode arrays where each ultra-narrowband photodiode corresponds to a specific monochromatic detection wavelength. Each ultra-narrowband photodiode consists of a conjugated-BIC photonic grating, a substitutable broadband semiconductor photodiode, and a combined waveguide connecting the grating and photodiode. Conjugated-BIC photonic gratings consisting of the patterned electron beam resist (ZEP520A) are fabricated on a tin oxide (SnO_2_) coated tin-doped indium oxide (ITO) glass substrate through electron beam lithography (EBL). When the light is projected onto the grating, the on-resonant monochromatic light will be extracted for in-plane propagation while the off-resonant light directly transmit to the free space. To minimize the attenuation of the coupled resonant light during in-plane propagation, a combined waveguide strategy is adopted without introducing additional waveguide materials and fabrication processes. Specifically, SnO_2_-coated ITO and ZEP520A serve as a combined waveguide in a whole, as well as the cathode and encapsulation for the photodiodes, respectively. In this case, the extracted monochromatic light will be coupled into the waveguide and propagate to the neighboring photodiode for signal output. Consequently, an ultra-narrowband photodiode has been formed. In this study, perovskite photodiodes are used as an example to demonstrate the compatibility of the proposed platform with novel solution-processed semiconductors. Scanning electron microscope (SEM) image of an ultra-narrowband perovskite photodiode is shown in Fig. 1b, where the zoom-in SEM image displays details of the conjugated-BIC grating. The overall size of the planarly-integrated device is around 100 × 150 μm and the height is within 1 μm, demonstrating the compactness and planarity. We also note that the grating serves as the area for light sensing in our system while the photodiode converts the resonant light into an optoelectronic signal. Although the fill factor (light sensing area versus photodetector pixel area) is not as high as 100%, this design is in agreement with the industrial standards like the pinned photodetectors in complementary metal oxide semiconductor sensors^27^.

Despite that several filterless narrowband mechanisms, which can potentially be used to construct spectrometers, have been reported in perovskites, our platform offers significant superiorities in constructing integrated spectrometers in terms of spectral resolution, wavelength tunability, device performance, and integration capability. We list and compare the proposed mechanism with the existing filterless narrowband mechanisms in perovskites (Fig. 1c). In the platform we proposed (upper panel), only monochromatic light with on-resonant wavelength, which can be facilely adjusted by tailoring the structure of the conjugated-BIC photonics, can be efficiently extracted for signal output, providing high spectral resolution, wavelength tunability, and high rejection ratio. For the conventional charge-collection narrowing (CCN) mechanism (middle panel), a narrowband response is enabled by the imbalanced carrier transit process generated by lights with different wavelengths in an optically and electrically thick perovskite film. While carriers generated by short-wavelength lights inherently recombined in the thick perovskite layer, carriers generated by long-wavelength lights can reach electrodes for the narrowband response. Incorporating a self-filter perovskite layer also filters short-wavelength lights while partially transmitting long-wavelength lights (lower panel), which is similar to that of the CCN mechanism. Although narrowband responses (linewidth: 10^1^–10^2^ nm scale) can be realized in the broadband-absorption perovskites employing the conventional narrowband mechanisms, the thick perovskite layer inevitably increases carrier diffusion length, which leads to deteriorative device performance. Besides, tediously changing the absorber thicknesses, compositions, and device structure in the photodetector arrays to realize wavelength tunability is also unfavorable for integration. Contrarily, our platform integrates perovskite photodiode arrays with one device configuration and appropriate perovskite thickness, which guarantees integration capability and device performance.

### Characterization of the conjugated-BIC photonic devices

**Fig. 2:**
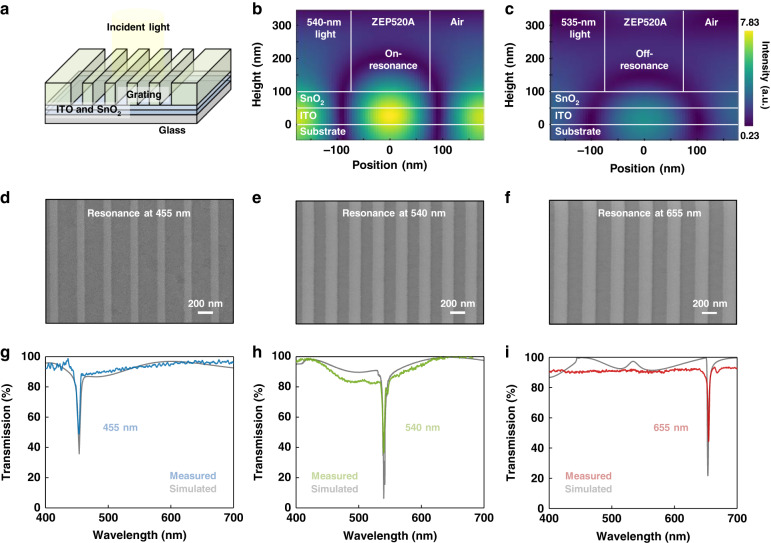
Resonance of the monochromatic lights. **a** Schematic model of the conjugated-BIC optical grating with the incident light projects onto the grating. The thicknesses of ITO, SnO_2_, and ZEP520A are set at 50 nm, 50 nm, and 350 nm, respectively. Field distribution at the grating with the **b** on-resonant light and **c** off-resonant light. Results show that the 540-nm on-resonant light generates a strong resonance with the grating, resulting in monochromatic light confinement. Top-view SEM images of the gratings with resonant wavelength at **d** 455 nm, **e** 540 nm, and **f** 655 nm. Results show that tuning the grating structure can effectively adjust the wavelength of the on-resonant light. Corresponding simulated and experimental transmission spectra of the gratings with resonant wavelength at **g** 455 nm, **h** 540 nm, and **i** 655 nm. On-resonant lights can be effectively confined and off-resonant lights will transmit through the grating, resulting in sharp dips in the transmission spectra

We systematically carry out both theoretical and experimental studies to reveal the functionality of the conjugated-BIC photonic gratings (see the schematic in Fig. 2a). Through the diffraction by the grating, on-resonant monochromatic lights can be confined and extracted for in-plane propagation. Therefore, the transmission of the on-resonant light will be suppressed, while the off-resonant light in other wavelengths transmits through the grating into the free space. Refractive indices (*n*) and extinction coefficients (*k*) of each layer consisting of the system are firstly characterized as the input parameter for the theoretical studies (Fig. S1). The optimal thicknesses of ITO, SnO_2_, and ZEP520A are then studied to guarantee maximum light coupling efficiency (Fig. S2). We also note that the grating period and grating width are used to adjust resonance at different wavelengths (Figs. S2 and S3).

In Fig. 2b, c, we present the cross-section field distribution of the 540-nm-resonant grating to illustrate the monochromatic light extraction process. The grating shows a strong resonance with 540-nm on-resonant light, leading to light confinement and local field enhancement (Fig. 2b). However, the 535-nm off-resonant light shows negligible interaction with the optical grating, resulting in light transmission into the free space (Fig. 2c). Figure 2d–f shows the SEM images of the optical grating for the three representative resonant wavelengths. Both experimental and calculated transmission spectra of the corresponding optical grating are shown in Fig. 2g–i, and transmission dips associated with the on-resonant wavelengths can be evident, indicating that the on-resonant monochromatic lights in the incident broadband lights are extracted for in-plane propagation without being transmitted. Besides, full-width at half-maximum (FWHM) of the three transmission dips are less than 4 nm, indicating that the coupled resonant lights are ultra-narrowband. Based on the measured linewidth and the absorption range of the perovskite photodiodes, transmission spectra of the conjugated-BIC photonic devices with resonant wavelengths from 400 to 700 nm (at 5-nm resonant wavelength interval) are characterized (Figs. S4 and S5) while the parameters of the gratings are also shown (Table S1).

### Characterization of the combined waveguide

**Fig. 3:**
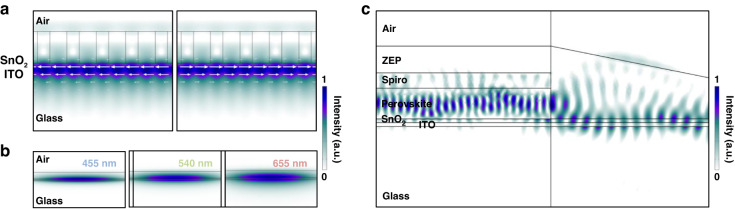
Propagation of the on-resonant monochromatic lights. **a** Propagation directions of the resonant light after being coupled into the waveguide. Results show that the resonant light will propagate perpendicularly with respect to the longitudinal direction of the grating strips in the in-plane direction. Arrows denote the propagation direction. **b** Field distribution in the waveguide with different monochromatic lights. Results show that different resonant lights can be effectively confined within the combined waveguide. **c** Light propagation at the perovskite-waveguide interface. Due to the large n of perovskites, resonant light propagating through the waveguide will further transport into the perovskite layer for an optoelectronic signal

As on-resonant monochromatic lights are extracted by the gratings, we then study the propagation of the monochromatic lights in the sequential waveguide. The propagation direction of the on-resonant light should be firstly controlled so that the light can reach the perovskite absorber. Figure 3a shows the simulated field distribution at the conjugated-BIC grating, and the direction of the field propagation is marked with arrows. Resonant light will perpendicularly propagate with respect to the longitudinal direction of the grating in the waveguide plane. In this case, the direction of resonant light propagation can be controlled by the rotation of the optical grating. Besides, the resonant light will propagate parallelly into two opposite directions, corresponding to two resonant modes^14,15^.

The loss of light during propagation should also be minimized to achieve an efficient optoelectronic output. However, using SnO_2_-coated ITO as the waveguide shows negligible light confinement and propagation ability (Fig. S6). By depositing ZEP520A with adequate thickness onto the SnO_2_-coated ITO substrate, a combined waveguide can be formed (Figs. S2 and S7). Figure 3b shows the simulated field distribution in the waveguide with three representative resonant wavelengths where the incorporation of ZEP520A effectively confines the light in the combined waveguide. Besides, the combined waveguide also significantly reduces the optical attenuation so that the extracted monochromatic lights can be effectively collected (Fig. S7 and Table S1). The morphology of SnO_2_ also plays an important role to the planar light propagation. SnO_2_ nanoparticles (NPs) are commonly used in perovskite optoelectronics due to their deposition simplicity^28–31^. However, SnO_2_ NPs can induce light scattering and lead to severe optical loss, which is evident by their high *k* (Fig. S1). By synthesizing a continuous SnO_2_ layer with smooth morphology, the light scattering can be effectively suppressed (Fig. S8). We further carry out studies to reveal how the SnO_2_ morphology affects the device performance. Results show that both spectral resolution and responsivity of the ultra-narrowband photodiode based on continuous SnO_2_ layer can be enhanced due to the suppression of light scattering (Fig. S8).

Due to the large *n* of perovskites (Fig. S1), on-resonant light propagates through the waveguide and can be effectively absorbed by the perovskite layer. The optimal thickness of the perovskite layer is also studied, which is a trade-off between the light coupling efficiency and the photodiode performance (Fig. S9). Here, theoretical calculations are adopted to show how light is absorbed into the perovskite in the integrated system. To better reveal the absorption of light, *k* is set to zero. As shown in Fig. 3c, resonant light can be effectively coupled into the perovskite layer for efficient optoelectronic conversion.

### Performance of the ultra-narrowband photodiodes

In this platform, both conjugated-BIC photonic devices and waveguides are fabricated on the exposed SnO_2_-coated ITO area next to the pre-fabricated broadband perovskite photodiode arrays. Due to the water and thermal sensitivity of the materials and interfaces in the perovskite devices, however, post-nanofabrications like lithography are rarely used. Herein, the EBL resist and resist developer are carefully selected so that both perovskite materials and devices are compatible with the nanofabrication processes (Figs S10–S14). In particular, the perovskite photodiodes work effectively under zero bias due to the low interfacial energy barriers (Fig. S13), indicating that the integrated spectrometers can be self-powered without power consumption.

**Fig. 4:**
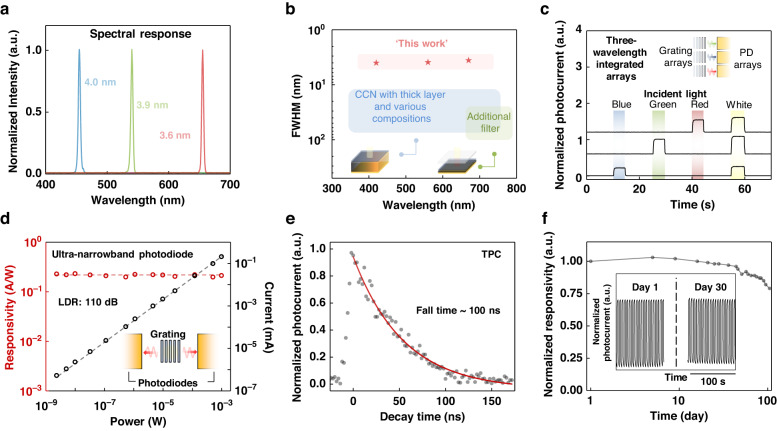
Ultra-narrowband perovskite photodiode characterizations. **a** Normalized spectral photocurrent of the ultra-narrowband perovskite photodiodes with three different resonant wavelengths. Spectra show that the spectral linewidth is as narrow as 3.6 nm. **b** Statistical spectral linewidth analysis of the state-of-the-art narrowband perovskite photodetectors. **c** Temporal response of the three-wavelength-integrated photodiode arrays. Results show that our ultra-narrowband photodiodes are capable to realize integration for light detection with different wavelengths. Note that incident light with different wavelengths is generated by different bandpass filters with continuous white light. Photocurrent magnitude difference results from the intensity disparity in different wavelengths that compose the continuous white light, as well as the attenuation of different lights in the waveguide. Inset, the schematic device structure of the multi-wavelength ultra-narrowband photodetector arrays. **d** The responsivity of the ultra-narrowband perovskite photodiode. The responsivity and LDR of our device reach 0.2 A W^–1^ and 110 dB, respectively, under 0 V bias and 720 nm incident light. Inset, the schematic device structure of the ultra-narrowband perovskite photodetector. **e** TPC measurement of the ultra-narrowband perovskite photodiode. The 100-ns fall time is significantly faster than the conventional perovskite narrowband photodetectors owing to the reduction of the carrier transit time. **f** Stability study of the ultra-narrowband perovskite photodiode. Due to the encapsulation of ZEP520A, the stability of our device is effectively enhanced

Firstly, the capability of distinguishing monochromatic light is studied. We note that the conjugated-BIC photonics serves as the light sensing area on which incident lights are directly projected for photocurrent measurements. Figure 4a shows the spectral response of the ultra-narrowband perovskite photodiodes with three representative on-resonant wavelengths, namely blue, green, and red. Owing to the conjugated-BIC photonics, spectral linewidth as narrow as 3.6 nm can be evident from our devices. Besides, the ultra-narrowband perovskite photodiodes also exhibit a rejection ratio as high as 125 (Fig. S15), and we attribute this high rejection ratio to the fact that the off-resonant lights directly transmit to the free space rather than being absorbed by the perovskite layer as that of the conventional perovskite narrowband photodetectors. We further compare the measured spectral linewidth with the reported values based on conventional narrowband mechanisms in perovskites to show that our devices possess an ultra-high spectral resolution and a low color artifact (Fig. 4b). Other than the three representative wavelengths, the spectral response of the ultra-narrowband perovskite photodiodes with on-resonant wavelengths ranging from 400 to 720 nm are characterized (Fig. S16), showing that our devices have a broad bandwidth. We note that the bandwidth is determined by the absorption range of the integrated broadband photodiodes, and the bandwidth can be effectively adjusted by substituting the semiconductors used in the photodiodes. To demonstrate the semiconductor substitutability in this platform, we fabricate ultra-narrowband photodiodes based on semiconductor quantum dots (QDs), and the results show that the ultra-narrowband QD photodiodes are also capable of differentiating monochromatic lights (Fig. S17).

Besides being capable of resolving light with narrow linewidth and broad bandwidth, the proposed ultra-narrowband perovskite photodiodes can also be facilely integrated into an array, which is favorable for fabricating integrated spectrometers. Figure 4c shows the temporal photocurrent of three-wavelength-integrated arrays under different illumination conditions, and the arrays are capable to resolve three different monochromatic incident lights as well as white light. This result is further supported by the optical images in Fig. S18.

Responsivity and detectivity of the ultra-narrowband photodiodes are also characterized to be as high as 0.2 A W^−1^ (Fig. 4d and Fig. S19a) and 8.5 × 10^11^ Jones (Fig. S19b, c), respectively. Another merit of our ultra-narrowband perovskite photodiode is the high response speed, which is measured by transient photocurrent (TPC) measurement. In general, the response speed of perovskite photodetectors can be expressed by the –3 dB bandwidth ${({f}_{-3\,{dB}})}^{-2}={(\frac{3.5}{2\pi {t}_{{tr}}})}^{-2}+{(\frac{1}{2\pi {RC}})}^{-2}$ where ${t}_{{tr}}$ is the carrier transit time and ${RC}$ is the resistance-capacitance time constant of the device^32^. In particular, the carrier transit time depicts the average time for a carrier to travel through a certain region. Unlike the thick perovskite layer used in the conventional perovskite narrowband photodetectors with the CCN mechanism, the thickness of the perovskite absorber is significantly reduced. Therefore, the carrier transit time in our system is also significantly reduced to accelerate the response to incident photons. As shown in Fig. 4e, our devices exhibit a response speed of around 100 ns, which is significantly faster than those of the conventional perovskite narrowband photodetectors with the CCN mechanism (usually in the scale of tens of µs). To better reveal the advantages of our ultra-narrowband photodiodes, we list the overall performance comparison between our device and the conventional perovskite narrowband photodetectors in Table S2. We also characterize the long-term stability of our BIC-integrated perovskite photodetectors (Fig. 4f). Our device exhibits a stable operation due to the encapsulation of ZEP520A, which is favorable for long-term applications.

### Performance of the integrated spectrometers

**Fig. 5:**
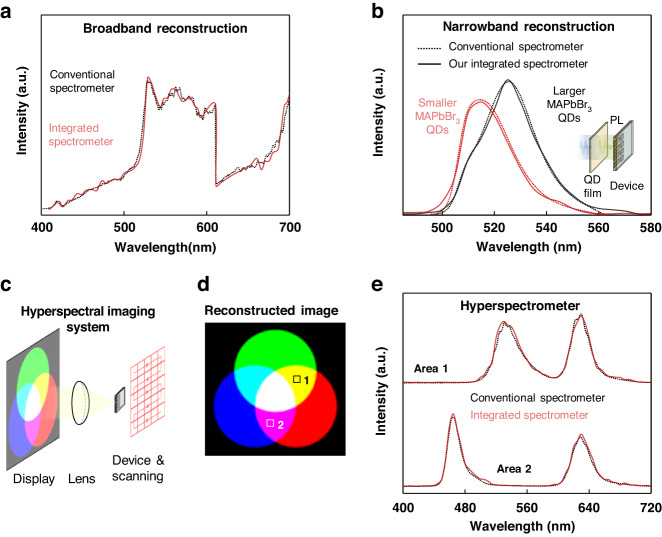
Spectral reconstruction and hyperspectral imaging. **a** Broadband and **b** narrowband spectral reconstruction using the integrated spectrometer. The broadband spectrum is generated by a continuous laser while the narrowband spectra are generated by the PL from QD film with different nanocrystal sizes. Results show that the integrated spectrometer is capable of precisely reconstructing input spectra. **c** Schematic measurement setup of the hyperspectral imaging. **d** Reconstructed RGB image and **e** spectral information of the two areas within. Results show that the integrated spectrometer can be used in hyperspectral imaging that resolves the spatial and spectral information

Given the performance of the ultra-narrowband photodiodes, integrated spectrometers based on the photodiode arrays are demonstrated. The spectrometer is composed of over 60 parallelly positioned perovskite photodiode arrays that are fabricated at once, and corresponding conjugated-BIC photonic devices with resonant-wavelength bandwidth that cover 400–720 nm (at 5-nm intervals). Herein, our integrated spectrometer is firstly applied to reconstruct the broadband spectrum of a white light. The spectrum reconstructed by our integrated spectrometer is shown to agree with that of the commercial spectrometer (Fig. 5a). Meanwhile, their capability to reconstruct narrowband spectra is also examined. In this study, the spectrometer is used to characterize the photoluminescence spectra of MAPbBr_3_ QDs. Lights emitted from QDs possesses narrow linewidth feathers and their wavelengths can be adjusted by tuning the size of QDs. Owing to the high spectral resolution, our integrated spectrometers are capable of differentiating lights emitted from the MAPbBr_3_ QDs with different sizes (Fig. 5b). The integrated spectrometers are operated without external power supplied since that the ultra-narrowband perovskite photodiodes are self-powered. This property can effectively reduce the power consumption for portable applications like point-of-care diagnosis and applications that integrated high density of sensors like internet-of-things.

Owing to its precise spectral reconstruction capability, hyperspectral imaging based on the integrated spectrometer is also demonstrated. Hyperspectral imaging collects point-to-point spectral information during the spatial and temporal imaging processes, which has emerged as an effective tool in remote sensing, medical, and military applications^33,34^. Specifically, integrated hyperspectrometers with the miniaturized feature are of interest in lab-on-chip applications like hand-held sensing, portable diagnosis, and all-optical information processing. In this study, an input RGB image generated by the display is firstly focused and projected onto the integrated spectrometer while the hyperspectral imaging is then achieved by scanning across the entire plane (Fig. 5c). The reconstructed RGB image agrees well with the input image (Fig. 5d). Our results show that the integrated spectrometer can be applied for precise spectral imaging. Specifically, the spectral information of two individual areas in the image is also characterized. The spectrum reconstructed by our integrated spectrometer matches well with that of the commercial spectrometer (Fig. 5e). Combined with the imaging functionality, our integrated spectrometer is capable to realize hyperspectral imaging.

## Discussion

Aiming at resolving the growing need of developing integrated spectrometers that are compatible with the multi-materials hetero-integration system in the point-of-care medical diagnosis, internet-of-things, and other lab-on-chip applications, this work proposes a facile and universal platform to fabricate wavelength de-multiplexing integrated spectrometers with semiconductor substitutability by unprecedently involving the conjugated-BIC photonics. Specifically, exploiting the conjugated-BIC photonics, which remains unexplored in conventional lasing studies, have demonstrated the opportunities in building ultra-narrowband photodiodes with ultra-narrow linewidth, broad/tunable bandwidth, high device performance, and on-chip integration ability, which is favorable for fabricating integrated spectrometers. The fabrication processes are also compatible with novel semiconductors photodiodes like perovskites and quantum dots, which can be potentially extended to conventional semiconductors. As an example, the integrated spectrometers based on ultra-narrowband perovskite photodiode arrays are capable of realizing narrowband/broadband light reconstruction and in-situ hyperspectral imaging.

## Materials and methods

### Materials

All materials used in this study are directly used as received, including methylammonium iodide (MAI, 99.9%, Greatcell solar), methylammonium bromide (MABr, 99.9%, Xi’an Polymer Light Technology), lead iodide (PbI_2_, 99.99%, Tokyo Chemical Industry), lead bromide (PbBr_2_, 99.9%, Xi’an Polymer Light Technology), cesium carbonate (Cs_2_CO_3_, 99.9%, Aladdin), N,N-dimethylformamide (DMF, anhydrous, Aladdin), dimethyl sulfoxide (DMSO, anhydrous, Aladdin), chlorobenzene (anhydrous, Aladdin), acetonitrile (ACN, anhydrous, Aladdin), amyl acetate (N50, anhydrous, Aladdin), isopropanol (IPA, anhydrous, Aladdin), acetone (anhydrous, Aladdin), toluene (anhydrous Aladdin), ethanol (anhydrous, Aladdin), 1-octadecebe (1-ODE, 90%, Aladdin), hexanes (anhydrous, Aladdin), methyl acetate (MeAc, anhydrous, Aladdin), oleic acid (OA, 85%, Aladdin), oleylamine (OAm, 70%, Aladdin), tin(II) chloride (SnCl_2_ ∙ 2H_2_O, 99.99%, Aladdin), tin(IV) oxide (SnO_2_, 15% in H_2_O colloidal dispersion, Alfa Aesar), spiro-MeOTAD (LT-S922, 99.5%, Lumtec), 4-tert-butylpyridine (tBP, Xi’an Polymer Light Technology), bis(trifluoromethylsulfonyl)amine lithium salt (Li-TFSI, Xi’an Polymer Light Technology), cobalt(III)Tris(bis(trifluoromethylsulfonyl)amine) (Co(III) TFSI, Xi’an Polymer Light Technology), 4-tert-butylpyridine (tBP, Xi’an Polymer Light Technology), tin-doped indium oxide (ITO, Henan Guluo Glass Co., Ltd), electron beam resist (ZEP520A, Zeon Co., Ltd).

### Device Fabrications

In this study, photodiode arrays are firstly fabricated, followed by the conjugated-BIC grating integration by electron beam lithography (EBL). In general, patterned ITO substrate (parallel ITO strips with 100-μm width and 100-μm interval) is cleaned by deionized water, IPA, acetone sequentially. ITO surface is further cleaned and activated by UV-ozone for 15 min before use. Then, a layer of SnO_2_ is fabricated on the ITO substrate. For compact SnO_2_, SnCl_2_ ∙ 2H_2_O solution (0.2 M in ethanol) is spun onto the ITO substrate (4000 rpm for 60 s), followed by annealing at 180 °C for 60 min. For SnO_2_ nanoparticles, SnO_2_ colloidal solution (dilute to 2.5% in water) is spun onto ITO substrate (1000 rpm for 30 s), followed by annealing at 150 °C for 30 min. The SnO_2_-coated ITO substrate is cleaned and activated by UV-ozone for 10 min before use. Perovskites or QDs are sequentially deposited onto the selected area of the SnO_2_-coated ITO substrate. Specifically, the surface of the substrate is partially covered by Kapton. After that, 70 µL of spiro-MeOTAD solution is dynamically spun onto the substrate. The spiro-MeOTAD solution is prepared by 72.5 mg spiro-MeOTAD, 17 µL Li-TFSI solution (260 mg Li-TFSI in 1 mL ACN), 30 µL tBP, 10 µL Co(III) solution (0.25 M in ACN), and 1 mL chlorobenzene. Eventually, 80 nm of gold is thermally evaporated as the top electrode to finish the fabrication of the perovskite photodetector. Scratches are made in between adjacent photodiodes to avoid crosstalk. Kapton should be removed from the substrate before the integration of BIC grating. Electron beam resist ZEP520A is then spun onto the entire substrate at 4000 rpm for 60 s and set in ambient condition overnight to fully evaporate the solvent. Electron beam lithography is carried out by Raith Eline 150Plus, followed by development with developer N50 for 60 s. For perovskite deposition, MAPbI_3_ solution (1.3 M) is prepared by dissolving stoichiometric MAI and PbI_2_ into DMF/DMSO mixed solvent (7:1). After that, MAPbI_3_ solution is then deposited onto the exposed area, followed by spin coating at 900 rpm for 5 s and 4500 rpm for 30 s. At the 5th s of the second spin coating stage, 0.2 mL chlorobenzene is deposited onto the substrate as the antisolvent. Finally, the substrate is annealed at 100 °C for 10 min. For QD deposition, QD solution is firstly synthesized. Specifically, 6.14 mmol Cs_2_CO_3_, 8 mL OA, and 100 mL 1-ODE are added into a 250-mL three-necked flask and the flask is kept at 90°C and vacuumed for 1 h. The flask is then filled with N_2_ and heated to 120 °C to obtain a clear Cs-oleate solution. Next, 2.16 mmol PbI_2_ and 50 mL 1-ODE are added into a three-necked flask and the flask is kept at 90 °C and vacuumed for 1 h. The flask is then filled with N_2_, followed by an injection of 5 mL OA and 5 mL OAm. After the dissolution of PbI_2_, a clear PbI_2_ solution can be obtained. The PbI_2_ solution is then heated to 160 °C, and 4 mL Cs-oleate solution under 100°C is quickly injected. The mixture is moved into an ice bath at the 5^th^ second after the injection. After cooling to room temperature, the mixture is transferred into the glovebox and 150 mL MeAc is added as the antisolvent. The mixture is then centrifuged at 8000 rpm for 5 min and the supernate is discarded. The QD precipitates are collected and dispersed in 18 mL hexane. To purify the QDs, 18 mL MeAc is added into the QDs in hexane solution, followed by centrifuge at 8000 rpm for 3 min to obtain the precipitates. The QD precipitates are then dispersed in 20 mL hexane and centrifuged at 4000 rpm for 5 min to obtain the supernate. The supernate (QD solution) is stored in the fridge overnight before use. To fabricate QD films, the QD solution is added onto the SnO_2_-coated ITO substrate, followed by spin coating at 3000 rpm for 30 s. To synthesize MAPbBr_3_ QD with different particle sizes for narrowband spectrum reconstruction, a simple ligand-assist reprecipitation method is adopted. Specifically, 0.1 mmol PbBr_2_, 0.1 mmol MABr, 50 μL OA, and 50 μL OAm are dissolved in 1 mL DMF. 100 μL of the solution is then added into 2 mL toluene under stirring to obtain MAPbBr_3_ QDs in the toluene solution. The particle size of the QDs can be adjusted by tuning the amount of OA and OAm. The MAPbBr_3_ QD film can be obtained by drop-casting the QD solution on a glass substrate.

### Characterizations

Transmission spectra of the BIC grating are carried out with a spectrometer (Ocean Optics Maya2000Pro) and a supercontinuum laser (NTK FUI-15). Characteristic I–V curves of the perovskite photodetectors without BIC grating are characterized by a source meter (Keithley 2636B) with a single-wavelength Thorlabs laser diode or a Zolix solar simulator. The external quantum efficiency of the device is measured by Zolix solar cell quantum efficiency system. Ultra-narrowband responses of the perovskite photodetectors are carried out using the same setup as the transmission spectrum measurement with an additional Keithley 2636B source meter. The responsivity of the photodetector is calculated by $R=I/P$ where ${R}$ is the responsivity, $I$ is the current, and $P$ is the illumination intensity. Specified detectivity is calculated by both ${D}^{* }=R\sqrt{A}/\sqrt{2q{I}_{d}}$ and $R\sqrt{A}/\sqrt{{I}_{{noise}}}$ where ${D}^{* }$ is the specified detectivity, $R$ is the responsivity, $A$ is the area of the device, $q$ is the elementary charge, ${I}_{d}$ is the dark current under a specific bias, and ${I}_{{noise}}$ is the noise current under a specific bias. The shot noise ${i}_{n,{s}}$, thermal noise ${i}_{n,{t}}$, and white noise ${i}_{n,{w}}$ are calculated ${i}_{n,{s}}={(2e{I}_{D}\varDelta f)}^{1/2}$, ${i}_{n,{t}}={(4{k}_{B}T\varDelta f/R)}^{1/2}$ and ${i}_{n,{w}}={({{i}_{n,{s}}}^{2}+{{i}_{n,{t}}}^{2})}^{1/2}$, respectively. $e$ is the elementary charge constant, ${I}_{D}$ is the dark current, $\varDelta f$ is the bandwidth, ${k}_{B}$ is the Boltzmann constant, $T$ is the measurement temperature, and $R$ is the differential resistance of the device at the measurement voltage. Transient photocurrent measurements are carried out following the published work with a Tektronix MSO54-2000 oscilloscope, a Tektronix AFG31152 waveform generator, and a single-wavelength Thorlabs laser diode. Surface morphology and roughness are characterized by an Asylum atomic force microscopy system. Refractive indices and extinction coefficients of different materials are characterized by a J.A. Woollam ellipsometry system. X-ray diffraction spectra are characterized by a Rigaku Smartlab system.

### Numerical simulations

All numerical simulations are carried out by the finite element analysis method following the published works^14,15^.

## Supplementary information

Supplementary Information for A platform for integrated spectrometers based on solution-processable semiconductors

## Data Availability

The data that support the findings if this study is available from the corresponding authors upon reasonable request.
