# Supplementary material for: A platform for integrated spectrometers based on solution-processable semiconductors
Source: Light Sci Appl. 2023 Jul 26;12:184. doi: 10.1038/s41377-023-01231-1 (PMC10368745; doi:10.1038/s41377-023-01231-1)
Supplement: Supplementary file 1 — Supplementary Information for A platform for integrated spectrometers based on solution-processable semiconductors [file 41377_2023_1231_MOESM1_ESM.docx]

Supplementary Information for

**A platform for integrated spectrometers based on solution-processable semiconductors**

Yanhao Li^1†^, Xiong Jiang^1†^, Yimu Chen^1†*^, Yuhan Wang^1^, Yunkai Wu^1^, De Yu^1^, Kaiyang Wang^1^, Sai Bai^2^, Shumin Xiao^1,3^, Qinghai Song^1,3*^

^1^Ministry of Industry and Information Technology Key Lab of Micro-Nano Optoelectronic Information System, Guangdong Provincial Key Laboratory of Semiconductor Optoelectronic Materials and Intelligent Photonic Systems, Harbin Institute of Technology (Shenzhen), Shenzhen, 518055, China.

^2^Institute of Fundamental and Frontier Sciences, University of Electronic Science and Technology of China, Chengdu, 611731, China.

^3^Collaborative Innovation Center of Extreme Optics, Shanxi University, Taiyuan, 030006, China.

^†^These authors contributed equally: Yanhao Li, Xiong Jiang, Yimu Chen.

^*^Corresponding authors: Yimu Chen (email: chenyimu@hit.edu.cn), Qinghai Song (qinghai.song@hit.edu.cn)

**Figure S1**

| 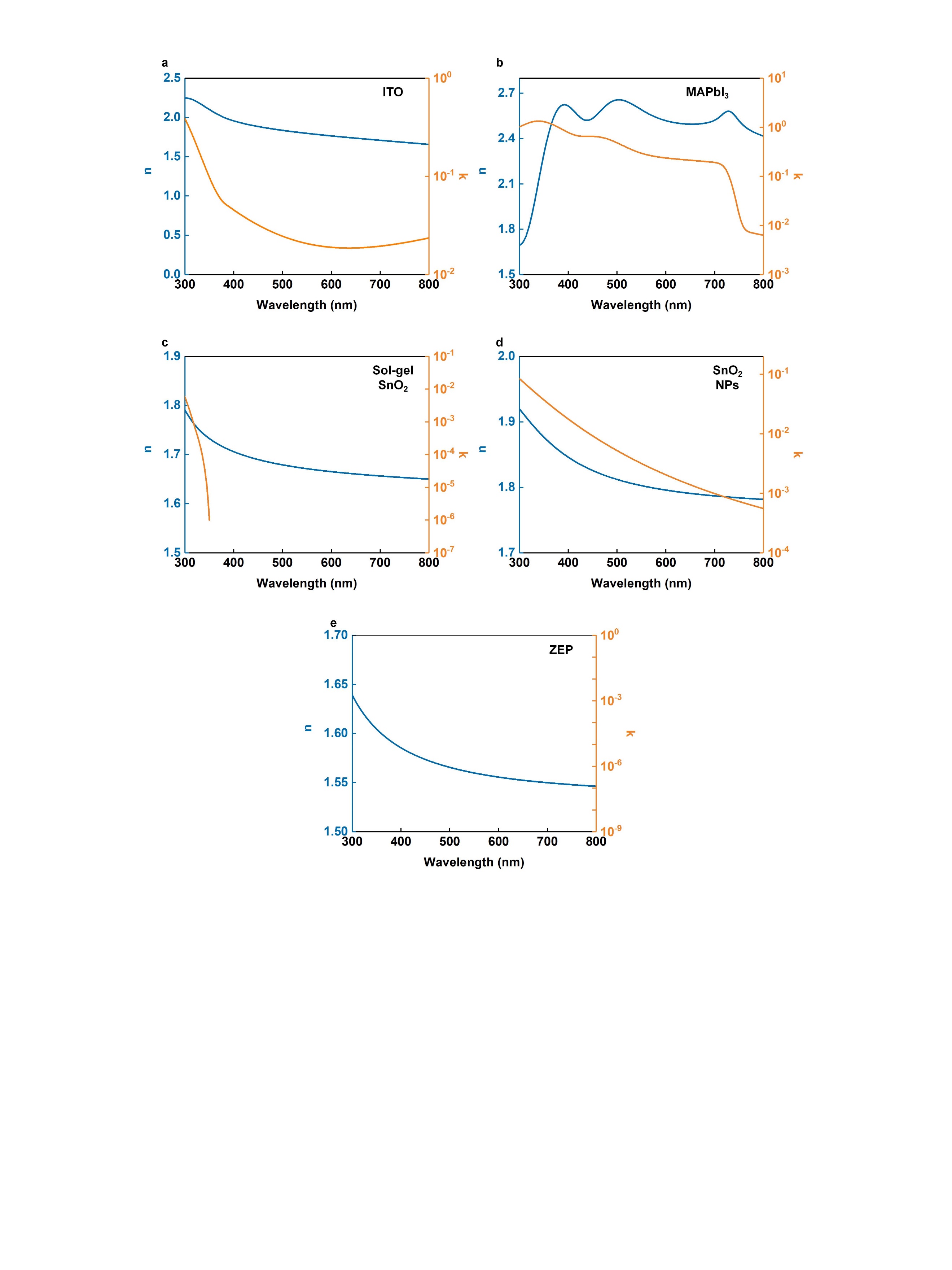 |
| --- |
| **Figure S1. Refractive indices (n) and extinction coefficients (k) characterizations.** (**a-e**) n and k of ITO, MAPbI_3_, sol-gel SnO_2_, SnO_2_ NPs, and ZEP520A, respectively. |

Finite element analysis simulations of the conjugated-BIC resonance and the resonant light propagation require highly accurate input refractive indices (n) and extinction coefficients (k) to generate reliable results. Therefore, we systematically characterize the n and k of the materials that we used in the simulations, including ITO, perovskite, ZEP520A, sol-gel SnO_2_ layer, and SnO_2_ nanoparticles (NPs). As shown in Figure S1, n of the perovskite is much higher than those of the ITO, SnO_2_, and ZEP520A. Therefore, photons in the combined waveguide can be effectively coupled into the perovskite layer when propagating and striking the perovskite photodetectors. Besides, k of the ITO can induce additional optical losses during light propagation (Figure S1a). Therefore, the thickness of ITO should be rationally reduced to minimize the optical losses while ensuring efficient device performance of the perovskite photodetectors. Note that sol-gel SnO_2_ thin film and ZEP520A show incomplete spectra of k (Figure S1c and S1e), which is due to the extremely low value that falls out of the measurement limit. In this case, the optical losses in the visible spectrum of these two kinds of materials will have a negligible impact on light propagation. k of SnO_2_ NPs thin film is much larger than that of the sol-gel SnO_2_ thin film, which further indicates the additional optical losses due to the light scattering (Figure S1c and S1d). Therefore, adopting sol-gel SnO_2_ thin film in our ultra-narrowband perovskite photodetectors can significantly reduce the optical losses for enhanced device performance.

**Figure S2**

| 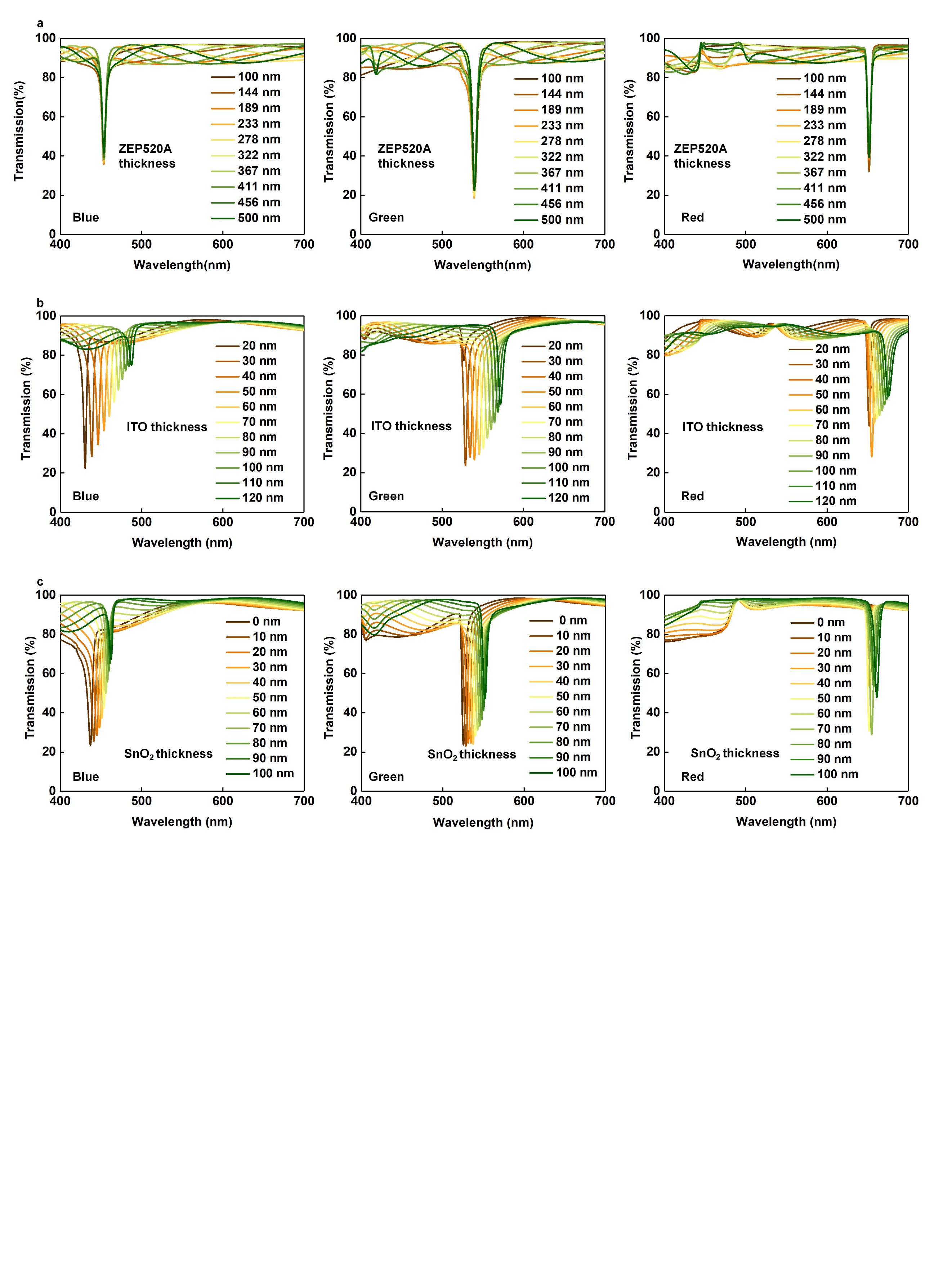 |
| --- |
| **Figure S2. Calculated transmission spectra of the conjugated-BIC photonics with different layer thicknesses.** (**a**) Transmission spectra as a function of the ZEP520A thickness. Within a reasonable range, the thickness of ZEP520A is found to have a trivial impact on the resonance. (**b**) Transmission spectra as a function of the ITO thickness. Reducing the thicknesses of ITO can increase the ratio of resonant light that is coupled into the waveguide for resonant lights at 540 nm and 455 nm. For 655-nm resonant light, reducing ITO thickness below 50 nm can fail in forming resonance mode. (**c**) Transmission spectra as a function of the SnO_2_ thickness. Otherwise mentioned, we use a combination of 350-nm ZEP520A, 50-nm ITO, and 50-nm SnO_2_ in the simulations. |

In this study, we propose to use integrated conjugated-BIC gratings to extract tunable monochromatic light for wavelength de-multiplexing. To understand how structural parameters of the gratings affect the light extraction process, we carry out simulations based on finite-element analysis. We specifically note that the selection of each grating parameter should also consider the light propagation and the device performance. The final parameter should be a trade-off among these three aspects. In particular, we systematically investigate the resonant wavelength and the efficiency of light coupling of the BIC grating by firstly evaluating the thickness of ZEP520A, SnO_2_, and ITO. Three representative resonant wavelengths, namely blue, green, and red, are investigated in this study. We find that the thickness of ZEP520A is relatively less important to the resonance as long as it is controlled within a reasonable range (100-500 nm), as shown in Figure S2a. Nevertheless, the thickness of ZEP520A can play a curial role in light propagation. Contrarily, the thicknesses of ITO and SnO_2_ can have a huge impact on the resonant wavelength and light coupling efficiency. Figure S2b and S2c show the correlation between the simulated transmission spectra and the thickness of ITO and SnO_2_, respectively. We find that reducing the thicknesses of both ITO and SnO_2_ can increase the ratio of resonant light that is coupled into the waveguide. However, it is impractical to change the resonant wavelength of the conjugated-BIC photonics by tuning the thickness of ITO and SnO_2_ since it is tedious during the fabrication of the integrated spectrometers. Therefore, the thickness of ITO and SnO_2_ should be optimized to a fixed value that considers the trade-off among the BIC resonant, optical loss, and device performance. For example, the reduction of SnO_2_ thickness can lead to light leakage during light propagation. Lowing the thickness of ITO and SnO_2_ can result in a severe increment of the series resistance and leakage current of the perovskite photodetectors, respectively. A thick SnO_2_ layer also blocks the carrier transport in the perovskite photodetectors and increases the series resistance. In this study, the thicknesses of ZEP520A, ITO, and SnO_2_ are optimized to be 350 nm, 50 nm, and 50 nm, respectively.

**Figure S3**

| 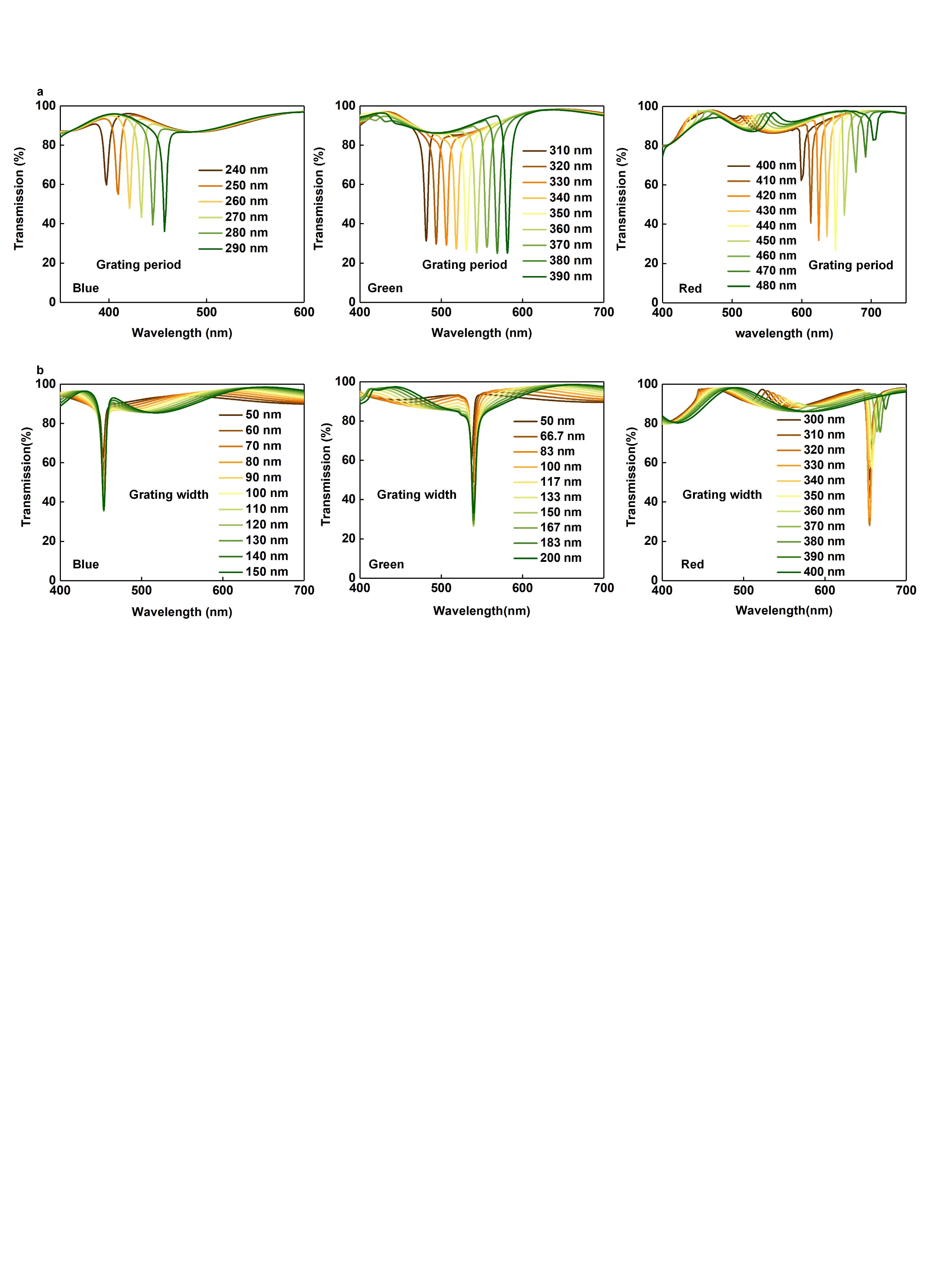 |
| --- |
| **Figure S3. Calculated transmission spectra of the conjugated-BIC photonics with different grating periods.** (**a**) Transmission spectra as a function of the grating strip width. Results show that altering the grating period can be an effective knob to change the resonant wavelength. (**b**) Transmission spectra as a function of the grating strip width. Being too narrow and too broad (approach grating period) can lead to the failure of forming a resonance mode. |

According to the content and discussion in Figure S2, we select to use the grating period and graing width as the knobs to tune the resonant wavelength of the conjugated-BIC photonics in the study. Results show that adjusting the grating period can effectively tune the resonant wavelength of the grating (Figure S3a). Although tuning the grating width cannot change the resonant wavelength, it plays an important role in ensuring coupling efficiency (Figure S3b). We also summarize the parameters of the grating period and grating strip width for the three representative resonant wavelengths we used in this study (Table S1). By the adjustment of the grating parameters, we can effectively tune the resonant wavelength of the grating for multi-wavelength ultra-narrowband photodetection. This strategy enables a facile approach to realize wavelength de-multiplexing for spectrometer applications. In particular, monochromatic lights can be effectively confined and extracted while their wavelengths can be easily tuned for broadband light reconstruction. In this case, a single broadband perovskite photodiode fabrication strategy for multi-wavelength on-chip integration can be used. This is fundamentally different from the conventional perovskite narrowband photodetectors based on the conventional charge-collection narrowing (CCN) mechanism and additional filters where perovskite photodetectors with different thicknesses, compositions, and additional self-filtering layers for multi-wavelength detection are extremely hard to be fabricated on a small area simultaneously for on-chip integration.

**Figure S4**

| 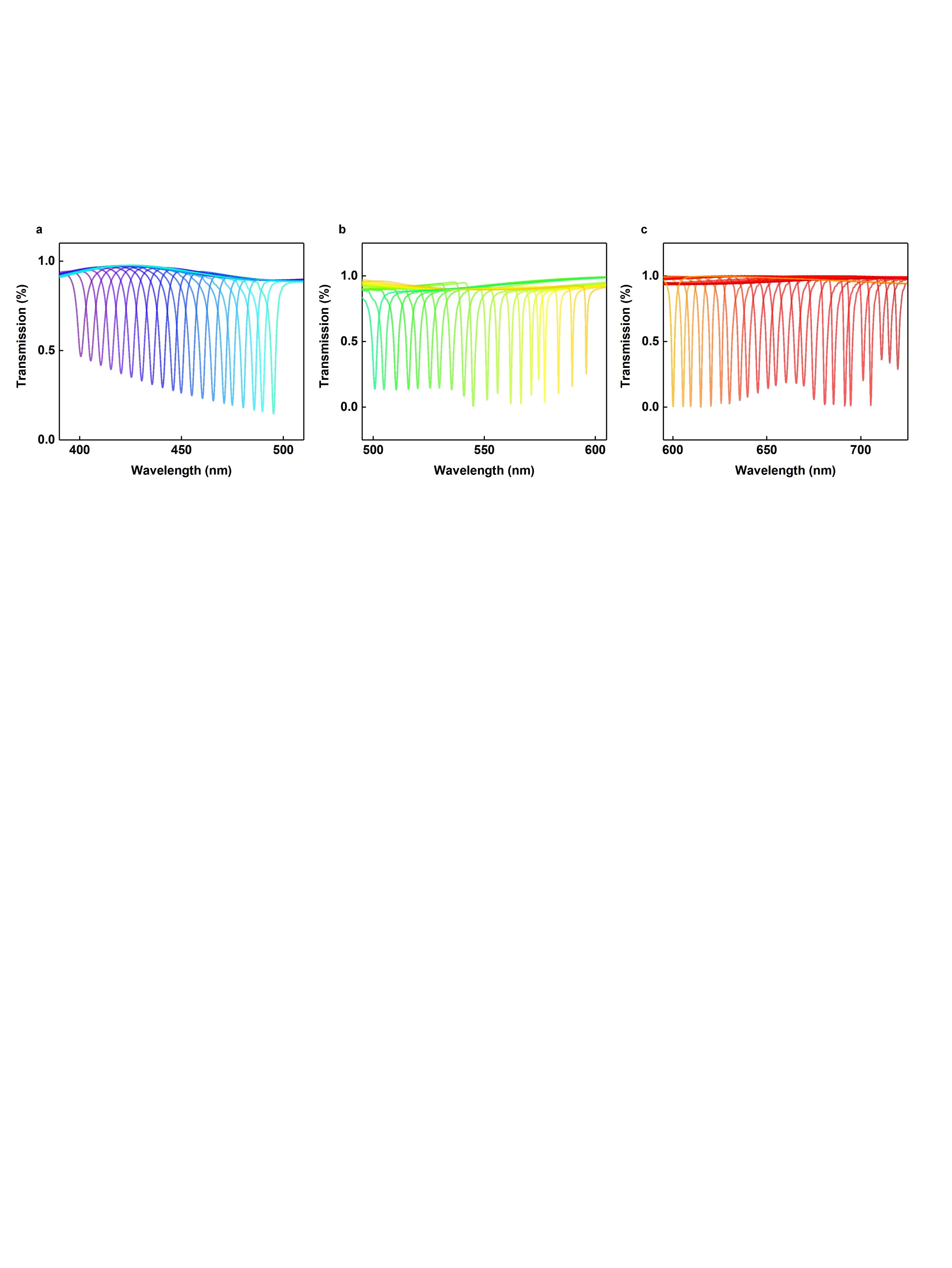 |
| --- |
| **Figure S4. Calculated transmission spectra of the conjugated-BIC photonics with different resonant wavelengths.** Calculated transmission spectra of conjugated-BIC photonics with the resonant wavelength from (**a**) 400-500 nm, (**b**) 500-600 nm, and (**c**) 600-720 nm. |

**Figure S5**

| 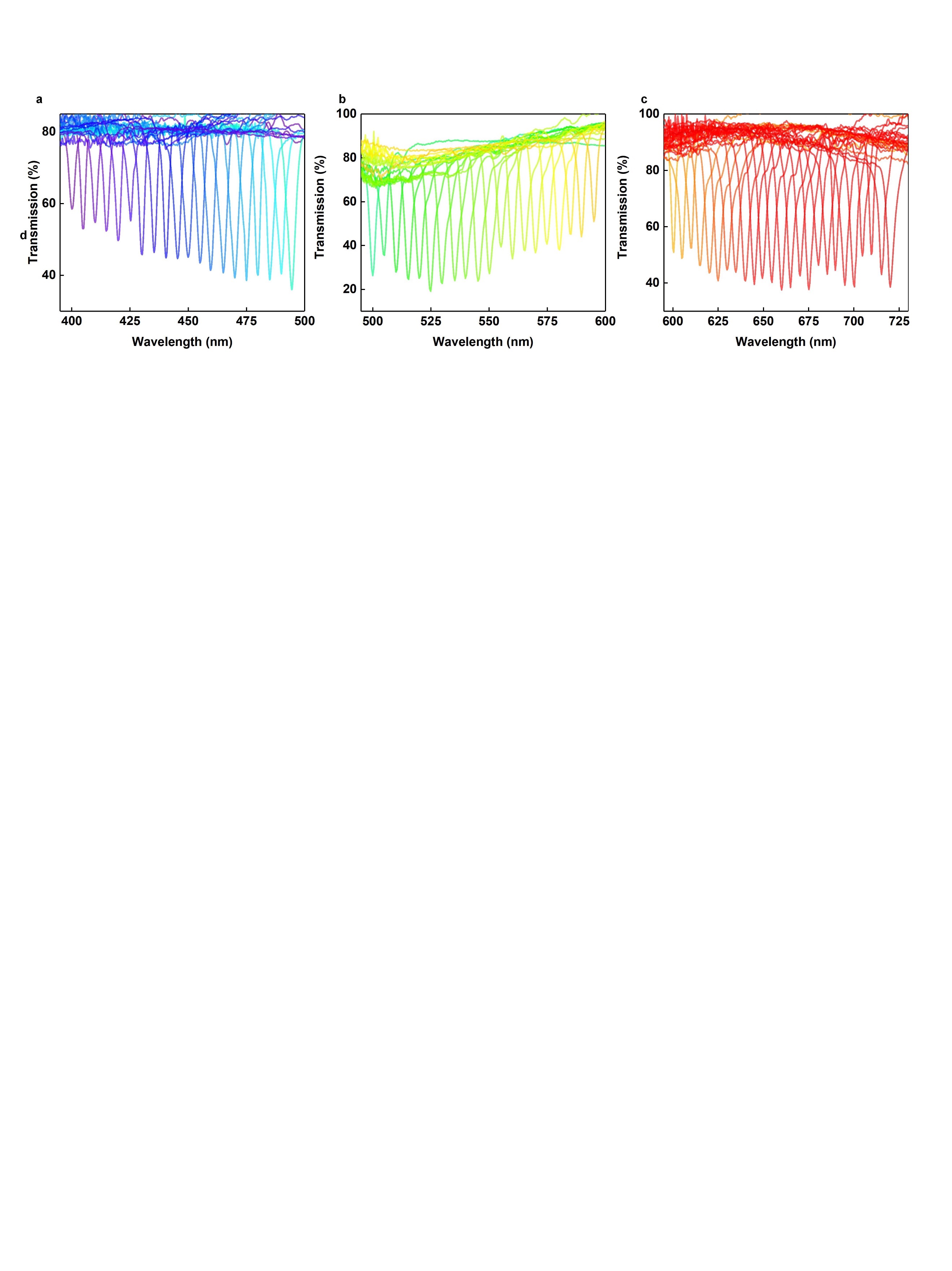 |
| --- |
| **Figure S5. Experimental transmission spectra of the conjugated-BIC photonics with different resonant wavelengths.** Transmission spectra of conjugated-BIC photonics with the resonant wavelength from (**a**) 400-500 nm, (**b**) 500-600 nm, and (**c**) 600-720 nm. |

**Figure S6**

| 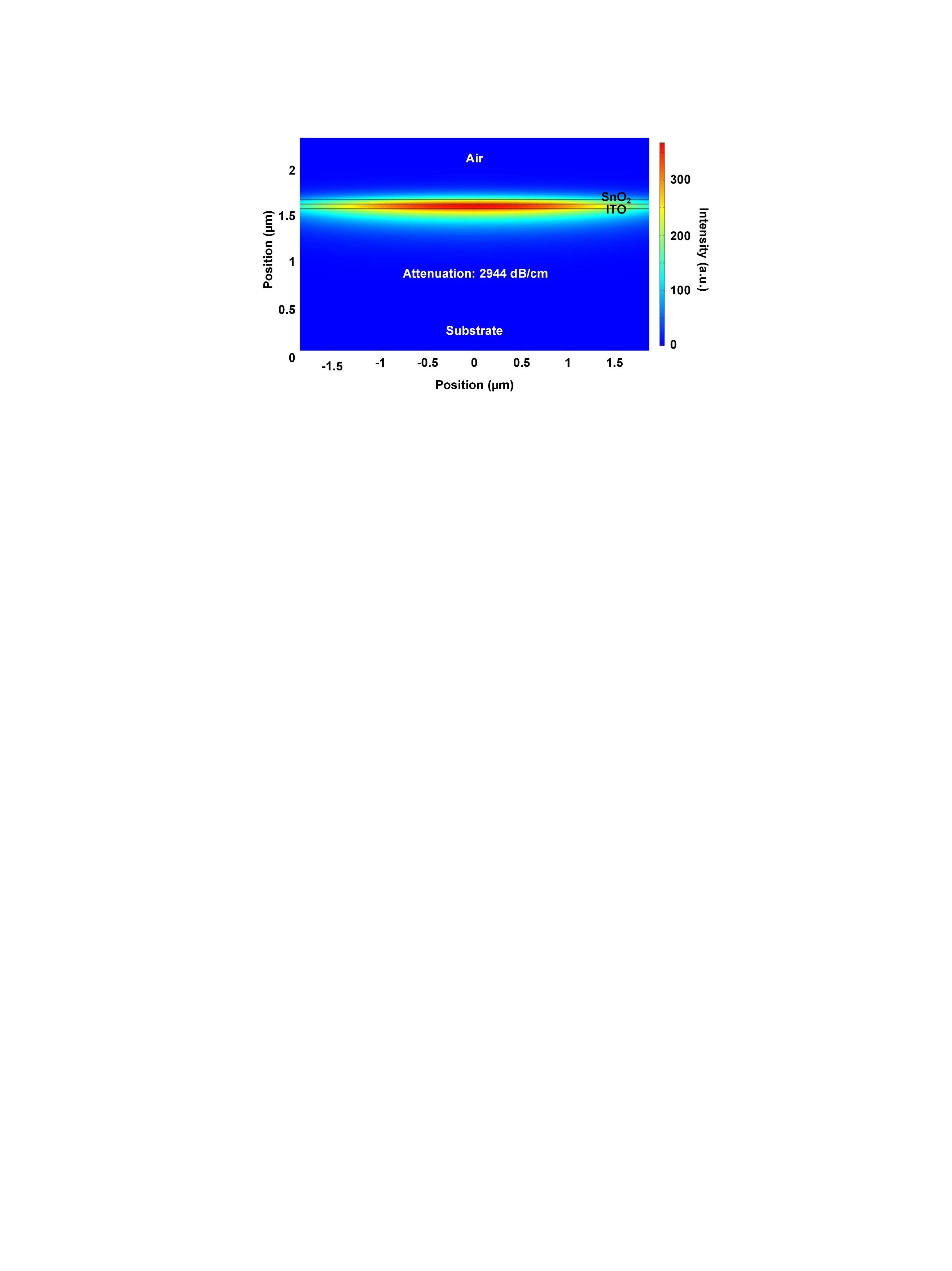 |
| --- |
| **Figure S6. Light propagation with ITO and SnO_2_ as the waveguide.** Field distribution of the waveguide cross-section reveals the optical loss (attenuation) reaches 2922 dB cm^-1^, which means that more than 49% of the coupled resonant light will be attenuated considering a 10-µm propagation distance. Meanwhile, most of the resonant light is restricted in the ITO layer, which further decreases the ratio of light that enters the perovskite photodetector. |

Controlling the optical losses during light propagation is considered one of the major challenges in this study. Using ITO and SnO_2_ in conventional perovskite devices as the waveguide is not practical for light propagation due to the limited thickness that results in the leakage and the large optical losses resulting from the large k of ITO. Figure S6 shows the field distribution in the waveguide consisting of ITO and SnO_2_. Results show that most of the resonant light will be confined in the ITO, leading to a high optical loss (attenuation) of over 2944 dB cm^-1^ due to the large k of ITO. This suggests that no effective waveguide mode can be formed. By integrating ZEP520A into the combined waveguide, light can be successfully confined in the ZEP520A and form a waveguide mode for light propagation (Figure 3b).

**Figure S7**

| 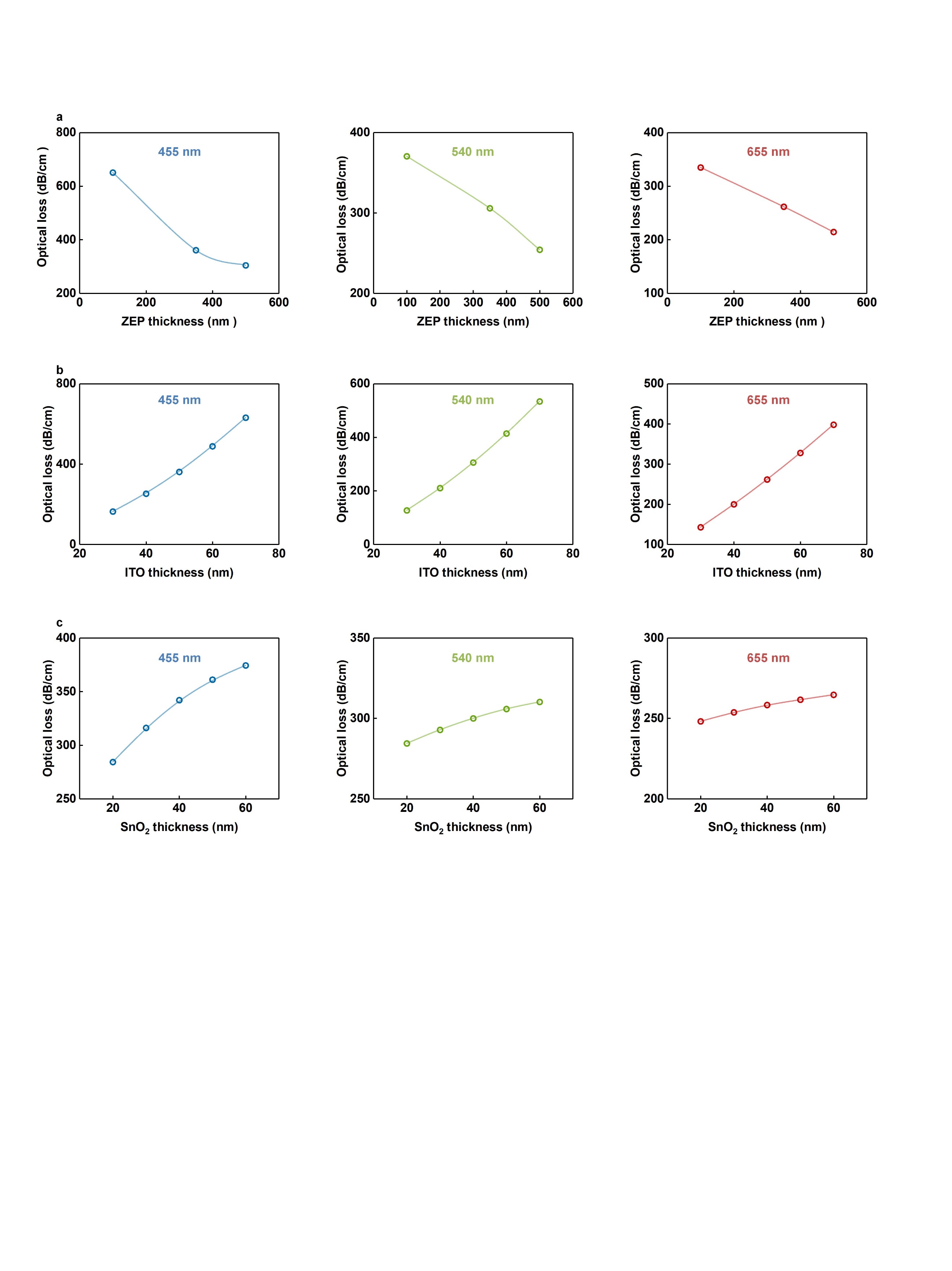 |
| --- |
| **Figure S7. Optical losses of the combined waveguides with different structural parameters.** (**a**) Optical loss as a function of ZEP520A thickness. Results show that increasing the ZEP520A thickness can effectively reduce optical losses. However, a 500-nm ZEP520A also increases the fabrication difficulty. (**b**) Optical loss as a function of ITO thickness. (**c**) Optical loss as a function of SnO_2_ thickness. Using thick ITO and SnO_2_ layers can lead to severe attenuation of the resonant light during propagation. |

To solve the light propagation challenges, we integrate the ZEP520A which consists of the BIC grating to form a combined waveguide with the SnO_2_-coated ITO. ZEP520A is a type of positive electron beam resists where the developer amyl acetate (N50) can dissolve the area exposed to the electron beam. Therefore, ZEP520A will cover the entire final device after all fabrication processes. In this case, ZEP520A can form a combined waveguide with the bare ITO and SnO_2_ in between the BIC grating and the perovskite photodetector. Resonant light can be confined in the combined waveguide with minimum leakage to the substrate during light propagation (Figure 3b). We systematically study the light propagation of three representative resonant wavelengths by varying the thicknesses of ZEP520A, ITO, and SnO_2_. In this study, the optical losses (attenuation) of the combined waveguide are adopted to evaluate the light propagation process. From the transmission spectra of the conjugated-BIC grating, varying the thickness of ZEP520A has a limited influence on the resonance (Figure S2a). However, a thin ZEP520A layer can lead to severe optical losses and leakage (Figure S7a). Besides, using a thick ZEP520A layer brings difficulties to the fabrication of the conjugated-BIC photonic gratings with narrow grating widths. Therefore, we adopt 350 nm for ZEP520A thickness in this study. We further investigate how ITO and SnO_2_ thickness affects the light propagation process. According to the thickness-dependent transmission spectra of ITO and SnO_2_ (Figure S2b and S2c), a rationally low thickness can significantly enhance the coupling ratio of the resonant light. Therefore, we carry out a thickness-dependent light propagation study based on the low-thickness range (30-70 nm for both ITO and SnO_2_). In terms of light propagation, adopting low ITO and SnO_2_ thicknesses induces extra light leakage to the substrate rather than confining light in the combined waveguide for all representative wavelengths. However, ITO thickness increment will lead to significant optical losses due to its large imaginary part (Figure S7b). Although SnO_2_ thickness increment will not lead to significant optical losses (Figure S7c), it can cause a severe series-resistance increment in the perovskite photodetectors. Based on these observations, we select 50-nm thick for both ITO and SnO_2_ in our following studies. In this case, a waveguide mode that minimizes the optical losses from over 2900 dB cm^-1^ to around 200 dB cm^-1^. Furthermore, the BIC gratings are integrated at around 10 µm from the edge of perovskite photodetectors. Therefore, the optical losses within such a short distance can be further suppressed. Through calculation, we find that over 95% of the resonant light can be collected if both sides of the grating along the resonant light propagation direction are integrated with photodiodes, considering a 10-µm combined waveguide.

**Figure S8**

| 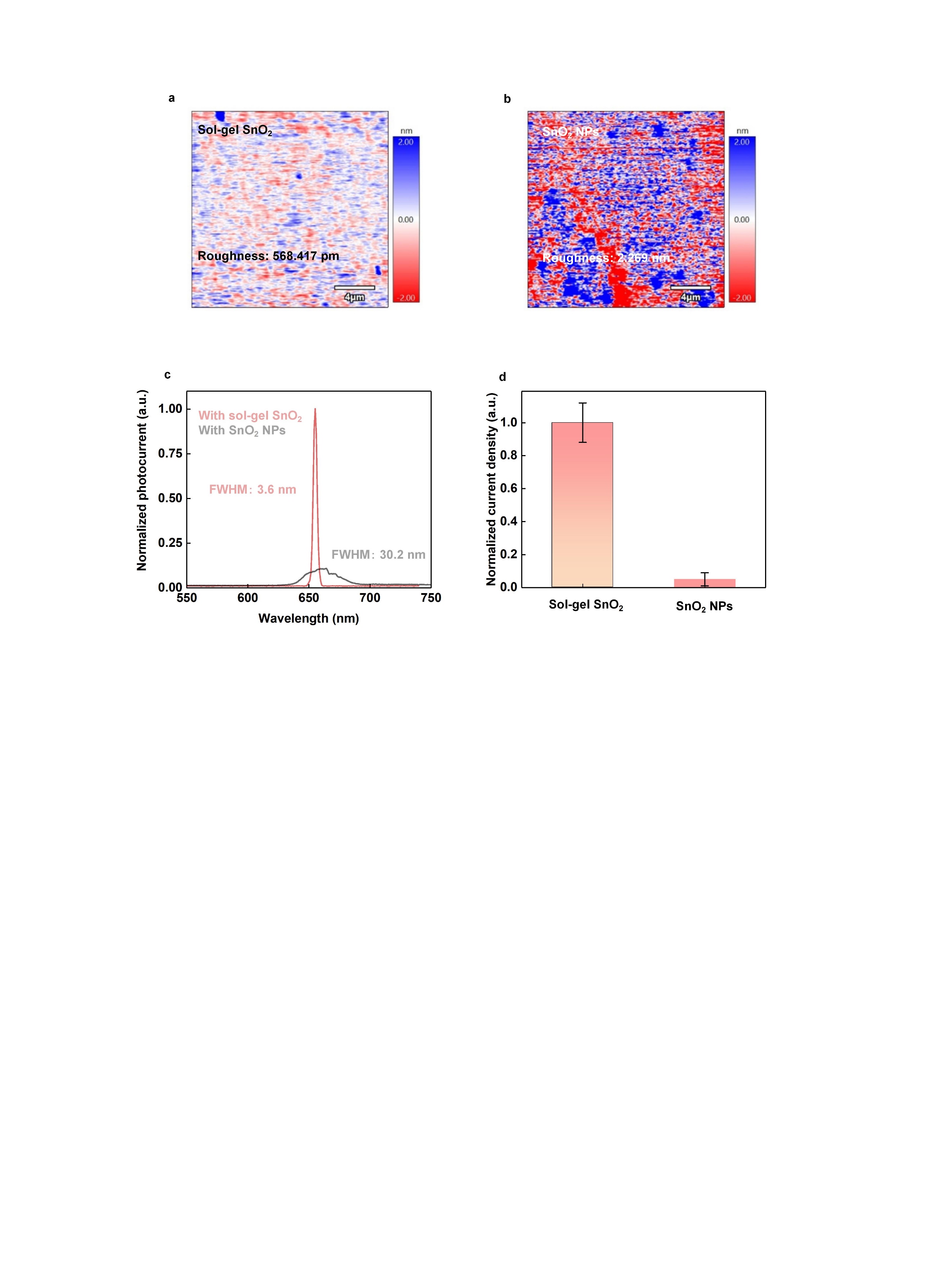 |
| --- |
| **Figure S8. Topographic study of SnO_2_ and the impact on the device performance. a**, **b**, Topography of the (**a**) sol-gel SnO_2_ thin film and (**b**) SnO_2_ NPs thin film. Results show that the sol-gel SnO_2_ thin film possesses a much-suppressed surface roughness (568.417 pm) than that of the SnO_2_ NPs thin film (2.269 nm), leading to severe light scattering and attenuation during the light propagation. (**c**) SnO_2_-morphology-dependent photocurrent of the ultra-narrowband perovskite photodetectors. Results show that the spectral photocurrent FWHM of the device based on the sol-gel SnO_2_ layer reaches 7.2 nm, which is much narrower than that of the device based on SnO_2_ NPs due to the suppressed photon scattering. (**d**) Statistical study of the photocurrent of the ultra-narrowband perovskite photodetectors using different SnO_2_ thin films. We note that the incident light is projected onto the optical grating for this study. Photodetectors with the sol-gel SnO_2_ thin films show an average photocurrent over one magnitude higher than those of the photodetectors with SnO_2_ NPs thin films. Number of experiments, n = 5 for each type of device. |

Optical losses due to the scattering during the propagation process can severely reduce the number of photons reaching the perovskite photodiodes, which is detrimental to the detection of optoelectronic signals. In the combined waveguide (ITO/SnO_2_/electron beam resist ZEP520A), the SnO_2_ layer plays a decisive role in the determination of the optical losses. In recent years, SnO_2_ NPs have emerged as an ideal electron transporting layer material in high-performance perovskite optoelectronics due to their deposition simplicity and high crystallinity[1]. However, their sub-wavelength particle size can lead to serious photon scattering, causing significant photocurrent decrement of the perovskite photodiodes. Therefore, we propose to synthesize a continuous layer of crystalline SnO_2_ by the sol-gel method[2]. Specifically, the sol-gel SnO_2_ layer is synthesized by depositing a layer of SnCl_2_·2H_2_O solution (in anhydrous ethanol), followed by annealing under the ambient condition at 180℃. Topographic studies of the sol-gel SnO_2_ and the SnO_2_ NPs indicate that the surface roughness of the sol-gel SnO_2_ (586.417 pm) is significantly reduced compared with that of the SnO_2_ NPs (2.269 nm) with the same thickness (Figure S8a and S8b). To further illustrate the photon scattering inhibition ability of the sol-gel SnO_2_ layer, we carry out photocurrent measurement in our ultra-narrowband perovskite photodiode systems with both sol-gel SnO_2_ layer and SnO_2_ NPs (Figure S8c). Note that the distance between the BIC grating and the perovskite photodetectors is kept identical to exclude the photon scattering due to the long traveling distance. Owing to the inhibition of photon scattering during the light propagation, the spectral response of the ultra-narrowband perovskite photodetector based on the SnO_2_ NPs layer shows an FWHM around 30.2 nm, which is much inferior to that of the device based on sol-gel SnO_2_ layer (as narrow as 3.6 nm). Besides, systems based on the sol-gel SnO_2_ layer exhibit a one-magnitude-enhanced photocurrent density (Figure S8d). Contrarily, the photocurrent density of the system based on the SnO_2_ NPs is greatly suppressed, which leads to the deterioration of the detection limit. These results correspond well with the n and k characterizations where the imaginary part of the SnO_2_ NPs is much larger than that of the sol-gel SnO_2_ thin film (Figure S1).

**Figure S9**

| 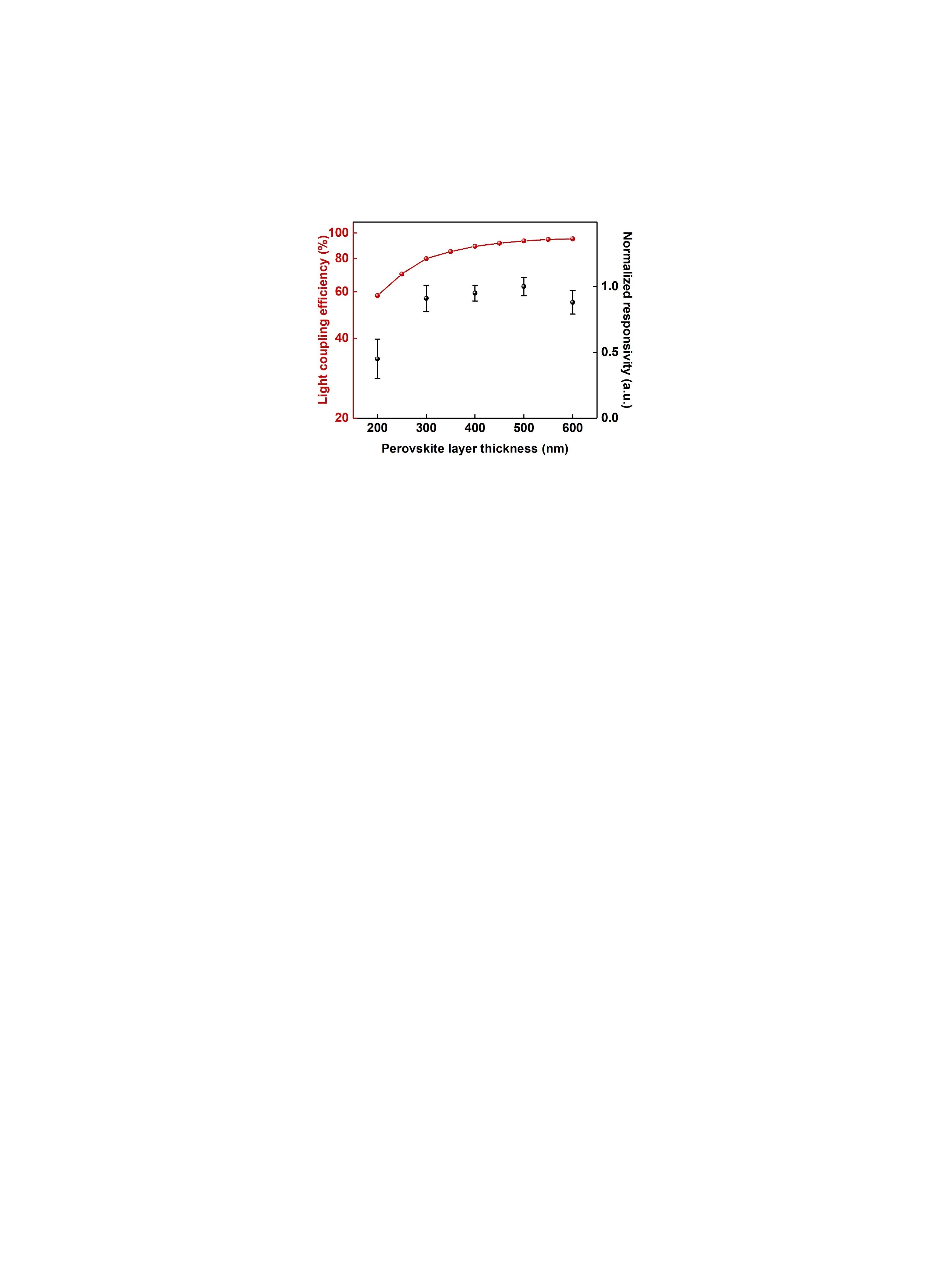 |
| --- |
| **Figure S9. Perovskite-thickness-dependent light coupling efficiency and responsivity.** With the increment of perovskite thickness, the light coupling efficiency can be enhanced. Meanwhile, the responsivity reaches the maximum value with a 500-nm-thick perovskite layer. Further increment of perovskite thickness induces severe non-radiative losses. |

In the proposed platform, the MAPbI_3_ perovskite layer thickness is set to be around 500 nm, which is a trade-off between the performance of perovskite photodetectors and the absorption of the resonant light. Considering the electrical resistance and the long distance for carriers to transport, further increasing the perovskite thickness can potentially lead to a drastic decrease in the optoelectronic performance although more resonant light can be coupled. Figure S9 shows the perovskite-thickness-dependent light coupling efficiency based on the finite element analysis. The ratio of light that is coupled to the device gradually increases with the increment of perovskite thickness. Due to the large refractive index of perovskite (Figure S1), resonant light can be effectively coupled into the perovskite. As we introduced above, however, the performance of the perovskite photodetector is also affected by the thickness of the perovskite layer. To reveal the trade-off between the light coupling efficiency and the performance of the photodetector, we characterize the perovskite-thickness-dependent responsivity based on the ultra-narrowband perovskite photodetector. As exhibited in Figure S9, we obtain the maximum responsivity with a 500-nm-thick perovskite. The responsivity gradually increases with the perovskite thickness increment and reaches a maximum value with the 500-nm-thick perovskite due to the improvement in the perovskite film coverage and defect density. However, further increasing the thickness of the perovskite layer leads to the acceleration of the non-radiative carrier recombination, resulting in the responsivity drop. Therefore, we adopt a 500-nm-thick perovskite thin film in our platform.

**Figure S10**

| 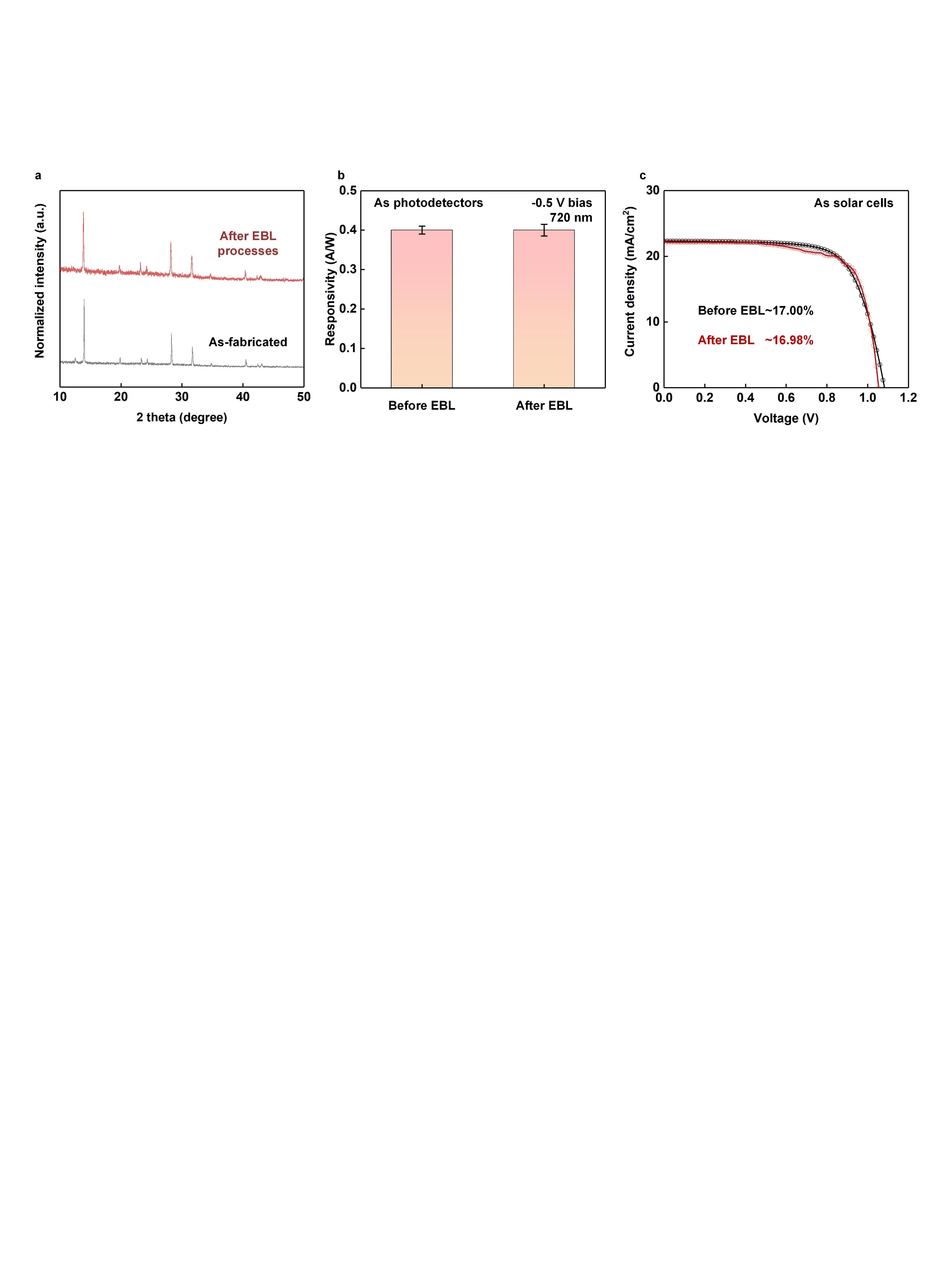 |
| --- |
| **Figure S10. Impact of the post-patterning EBL processes on the perovskite materials and devices.** (**a**) XRD spectra of the perovskite before and after the post-patterning process. XRD spectra show that no decomposition of the perovskite is observed after the EBL process, indicating that the EBL patterning and the electron beam resist ZEP520A are safe for perovskite materials. We note that a layer of ZEP520A is directly coated onto the perovskite in this study. (**b**) The statistical responsivity of perovskite devices as photodetectors. Results show that the EBL patterning process shows a negligible influence on the perovskite devices. The measurements are carried out under -0.5 V bias and 720-nm incident light. Number of experiments, n = 5. (**c**) Current density-voltage curves of perovskite devices as solar cells before and after the post-patterning process, demonstrate the same conclusion as that of (**b**)**.** |

Integration of perovskite materials and devices has always been a hot topic in the perovskite community since many modern electronics set a high standard in miniaturization and multi-functionalization[3-6]. Post nano/micro-fabrication of perovskite materials and devices is rarely studied in the field due to the water sensitivity of this class of materials. In this study, BIC grating is fabricated by EBL and post-development of the electron beam resist. Meanwhile, the electron beam resist also serves as part of the waveguide for light transportation. Therefore, it is crucial to ensure that this post-fabrication process is harmless to the perovskite materials and the performance of photodetectors. We carefully select ZEP520A as the electron beam resist for this study. ZEP520A is reported to be harmless to the perovskites and suitable for fabricating waveguides and grating with perovskite materials[5, 7]. However, it is unknown to the field whether ZEP520A will deteriorate the performance of perovskite diode devices with additional carrier transporting layers. Therefore, we conduct a systematic study to characterize the material properties and device performance before and after the nano-fabrication processes, including EBL patterning and wet-process development. Before the device performance characterizations, we firstly study the material properties before and after the nano-fabrication processes. A layer of ZEP520A is deposited onto the MAPbI_3_ thin film by spin coating and set under room temperature without heating overnight to fully evaporate the solvent in ZEP520A while avoiding perovskite decomposition due to long-time annealing. After that, the sample undergoes an EBL patterning process and development process using Amyl acetate (N50) as the developer. Figure S10a shows the X-ray Diffraction spectra of the as-fabricated MAPbI_3_ thin film and MAPbI_3_ thin film after the deposition of ZEP520A. No additional diffraction peak can be observed from the MAPbI_3_ thin film covered by ZEP520A, indicating that the EBL patterning and the ZEP520A itself will not decompose the perovskite materials. Therefore, we can conclude that the MAPbI_3_ thin film can be safely protected during the post-fabrication processes. We attribute the slightly higher noise level of the upper curves to the encapsulation of ZEP520A layer. The impacts of the nano-fabrication processes on the device performance are also studied. Specifically, we characterize the perovskite diodes as both photodiodes and solar cells. In this study, the as-fabricated perovskite photodiodes are coated with ZEP520A and set under room temperature for overnight to fully evaporate the solvent in ZEP520A. The devices then undergo the nano-fabrication processes without removing the ZEP520A. This is because the hole-transporting layer can be dissolved by the chlorobenzene during the ZEP520A removal process. We note that the incident light is projected from the back of the transparent ITO glass in these studies. The responsivity of the photodiode is firstly measured under a -0.5 V reversed bias and a 720 nm illumination condition (Figure S10b). Under the same illumination intensity, the responsivities of the as-fabricated and the post-fabricated photodetectors exhibit a negligible difference, demonstrating that the nano-fabrication processes will not damage the performance of the perovskite devices. We then characterize the perovskite diode as solar cells to further illustrate this conclusion (Figure S10c). Similar to the responsivity, the power conversion efficiency (PCE) of the photodetector barely changes after the post-fabrication processes, further demonstrating that the EBL patterning processes are harmless to the device performance. Therefore, it is safe to conclude that the grating-integration process by the EBL post-fabrication is suitable for perovskite electronics, which paves the road for perovskite electronics on-chip integration.

**Figure S11**

| 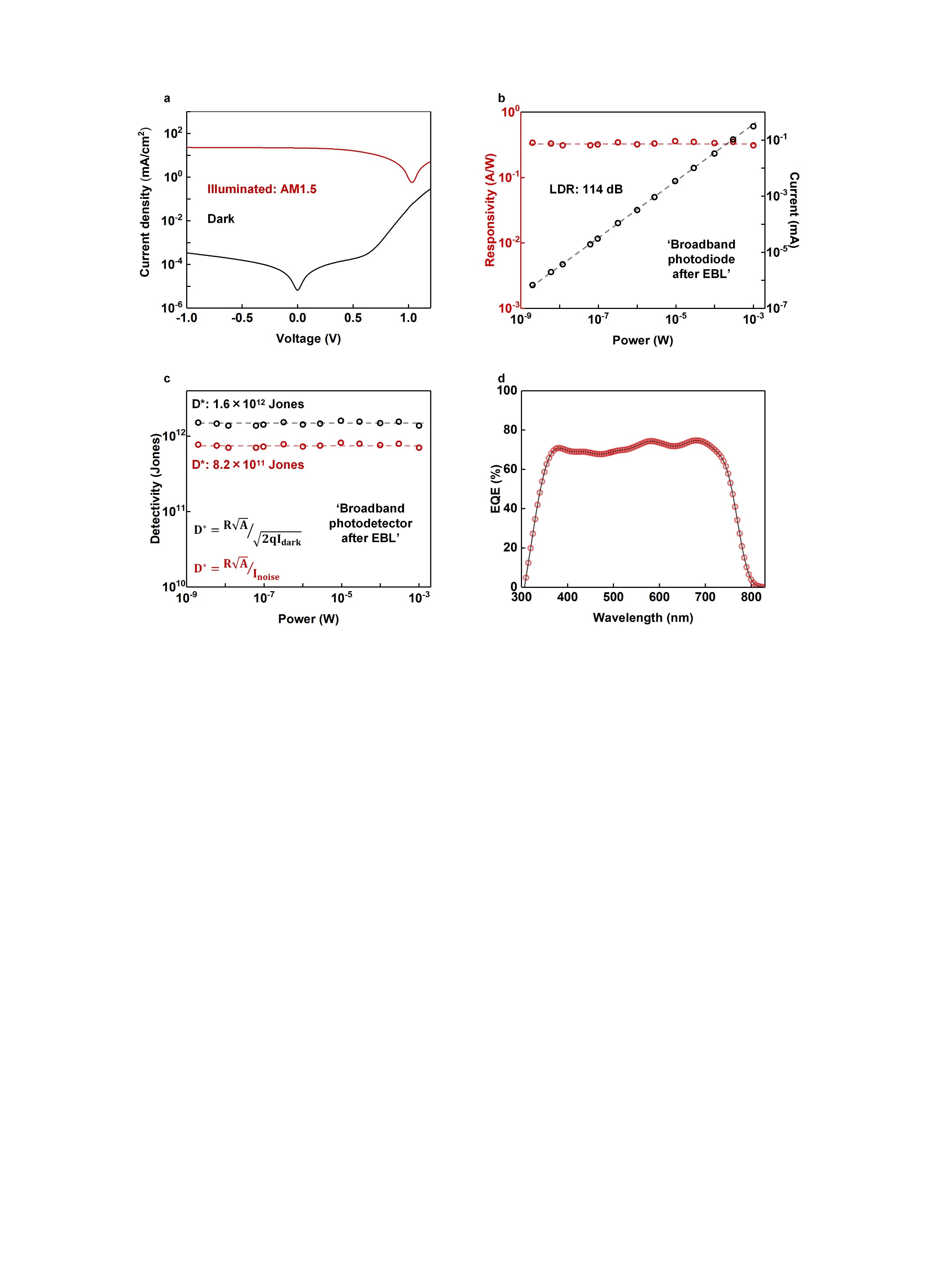 |
| --- |
| **Figure S11. Detailed characterization of photodiodes after EBL patterning process.** (**a**) Characteristic I-V curves of the post-patterning perovskite photodiode under dark conditions and illumination. Under the AM 1.5 illumination condition, the post-patterning perovskite photodetector shows a decent photocurrent and a large open-circuit voltage shift, showing that the EBL patterning process is safe for perovskite optoelectronics. (**b**) Responsivity and photocurrent of the post-patterning perovskite photodiode as a function of the illumination intensity. Note that the laser beam incident from the back of the substrate has an area smaller than that of the Au electrode. The measurement is carried out under -0.5 V bias with a 720-nm incident light. (**c**) Specified detectivity of the perovskite photodiode. Results show that the specified detectivity of our device reaches 8.7×10^11^ Jones, which is on par with the reported perovskite narrowband photodiode and demonstrates that the post-fabrication process and thin ITO are of minor influence on our system. (**d**) External quantum efficiency (EQE) of the post-patterning perovskite photodiode indicates that the post-patterning perovskite photodiode maintains an efficient photon conversion efficiency. We note that the measurements are carried out with light incident from the back of the ITO substrate and the device area is 1 mm by 1 mm. |

Micro/nanofabrication techniques like lithography are rarely studied in perovskite optoelectronics due to the water and thermal sensitivity of perovskites, carrier transporting materials, and interfaces. Contrarily, our perovskite photodiode presents compatibility with the EBL post-fabrication processes, and exhibits decent performance due to the careful selection of EBL resist and developer. Dark and illuminated characteristic I-V curves indicate that the device is well-protected by the ZEP520A during the EBL patterning process (Figure S11a). We then measure the responsivity and photocurrent density as a function of the illumination intensity under -0.5 V bias. As shown in Figure S11b, our device exhibits a responsivity around 0.42 A/W and a linear dynamic range over 114 dB. A statistical performance comparison between the perovskite photodetector before and after the EBL post-fabrication processes further indicates that the perovskite and device are compatible with the EBL post-fabrication processes (Figure S9). Besides, the noise current and the specified detectivity of the photodetector are also characterized. The noise current is determined to be 4.4×10^-14^ A Hz^-0.5^ (Figure S12), while the specified detectivity calculated with the noise current reaches 8.2×10^11^ Jones (Figure S11c), showing the capability of low-limit detection. EQE of the photodetector also reaches 75% (Figure S11d). These results show that our post-nanofabrication strategy can be applied in fabricating high-performance perovskite optoelectronics.

**Figure S12**

| 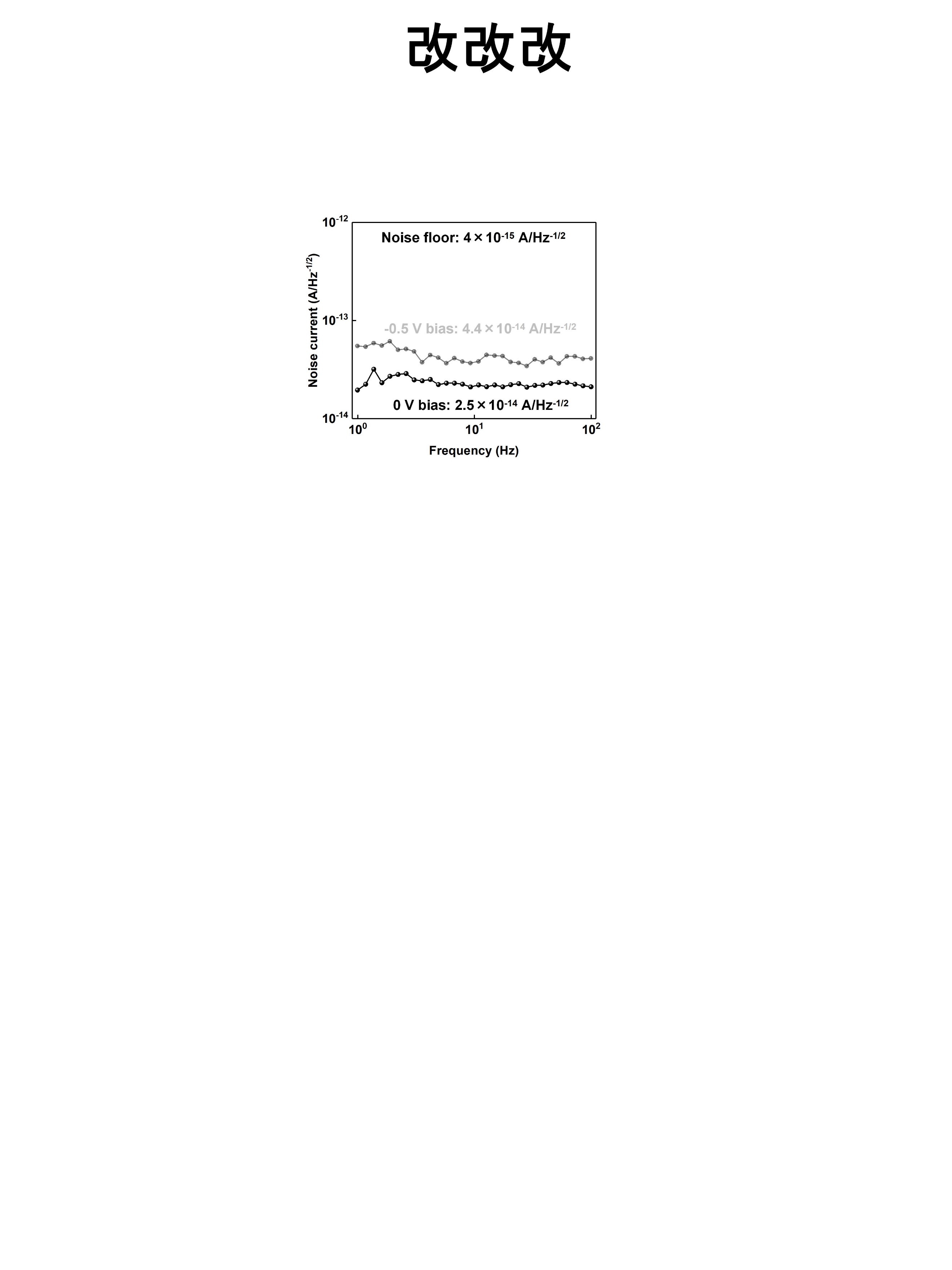 |
| --- |
| **Figure S12. Noise current measurement of the perovskite photodiode.** The noise current is determined to be around 2.5×10^-14^ and 4.4×10^-14^ A Hz^-0.5^ under 0 and -0.5 V bias, respectively. The noise current is less-dependent on the frequency, indicating that the flicker noise is negligible, and the white noise dominates the noise current[8-12]. |

In perovskite photodiodes, the total noise current is composed of white noise (shot noise and thermal noise) and flicker noise**.** The shot noise and thermal noise can be directly calculated based on the dark current measurement. Specifically, shot noise $i_{n, s}={(2eI_{D}\Delta f)}^{1/2}$, thermal noise $i_{n, t}={(\frac{4k_{B}T\Delta f}{R})}^{1/2}$, and white noise $i_{n, w}={({i_{n, s}}^{2}+{i_{n, t}}^{2})}^{1/2}$, where $e$ is the elementary charge constant, $I_{D}$ is the dark current, $\Delta f$ is the bandwidth, $k_{B}$ is the Boltzmann constant, $T$ is the measurement temperature, and $R$ is the differential resistance of the device at the measurement voltage. For the device under -0.5 V bias, the shot noise, thermal noise, and white noise are calculated to be 2.25×10^-14^ A Hz^-0.5^, 7.43×10^-15^ A Hz^-0.5^, and 2.37×10^-14^ A Hz^-0.5^, respectively. For the device under 0 V bias, the shot noise, thermal noise, and white noise are calculated to be 5.66×10^-15^ A Hz^-0.5^, 7.52×10^-15^ A Hz^-0.5^, and 9.34×10^-15^ A Hz^-0.5^, respectively. The flicker noise, which is also known as 1/f noise, shows a high dependency on the measurement frequency. In Figure S12, we show that the noise current of our device is less-dependent on the measurement frequency, indicating that the flicker noise is negligible in our device. Therefore, the white noise, which is composed of shot noise and thermal noise, dominates the total noise current and the calculated white noise value agrees well with the measured value in Figure S12.

**Figure S13**

| 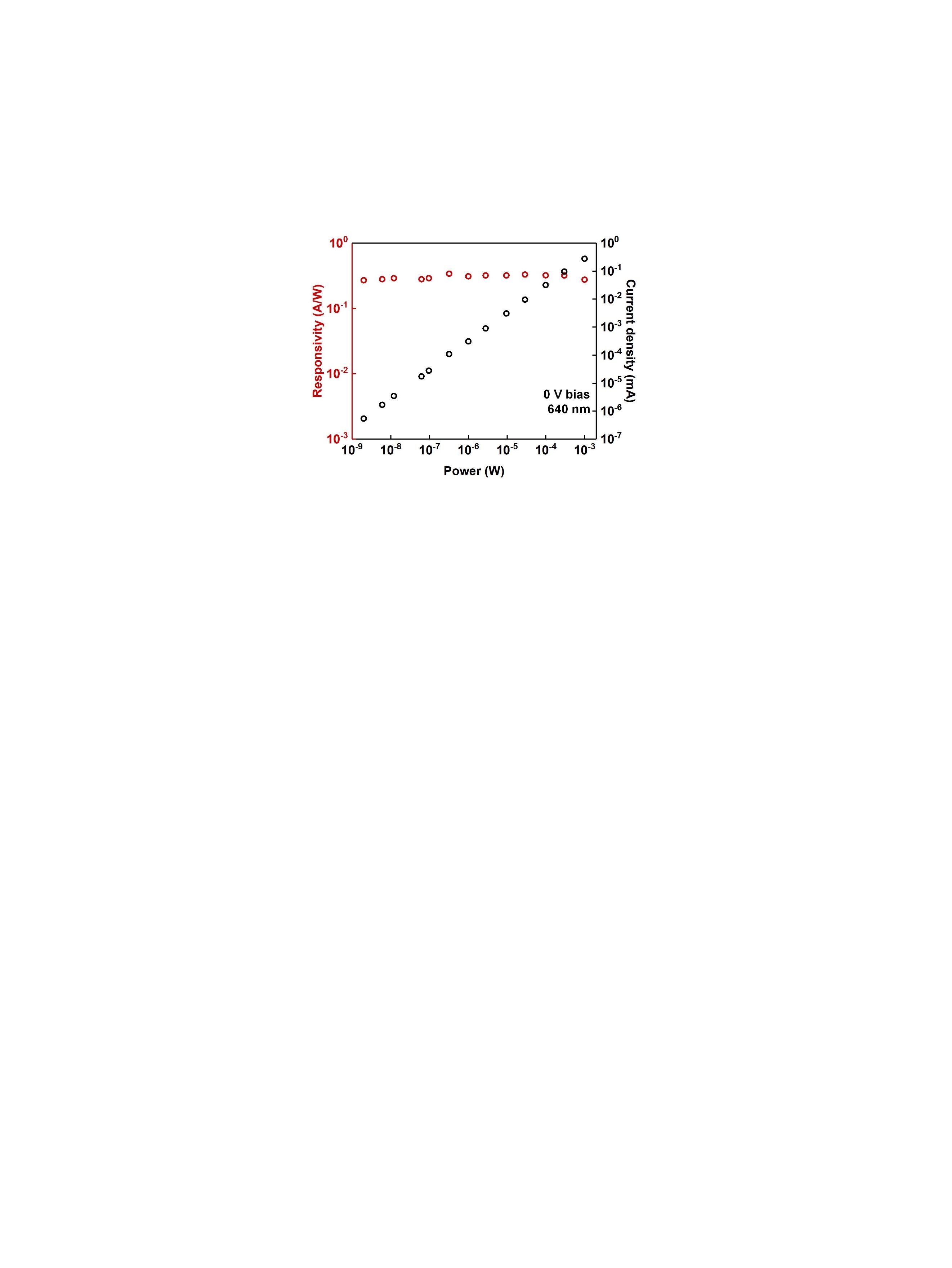 |
| --- |
| **Figure S13. Perovskite photodetector characterization under 0 V bias.** The responsivity under 0 V bias shows a marginal decade when compared with that of -0.5 V bias, indicating that the interfacial energy barriers are low in the device and the interfaces are well-protected during the post-fabrication processes. |

**Figure S14**

| 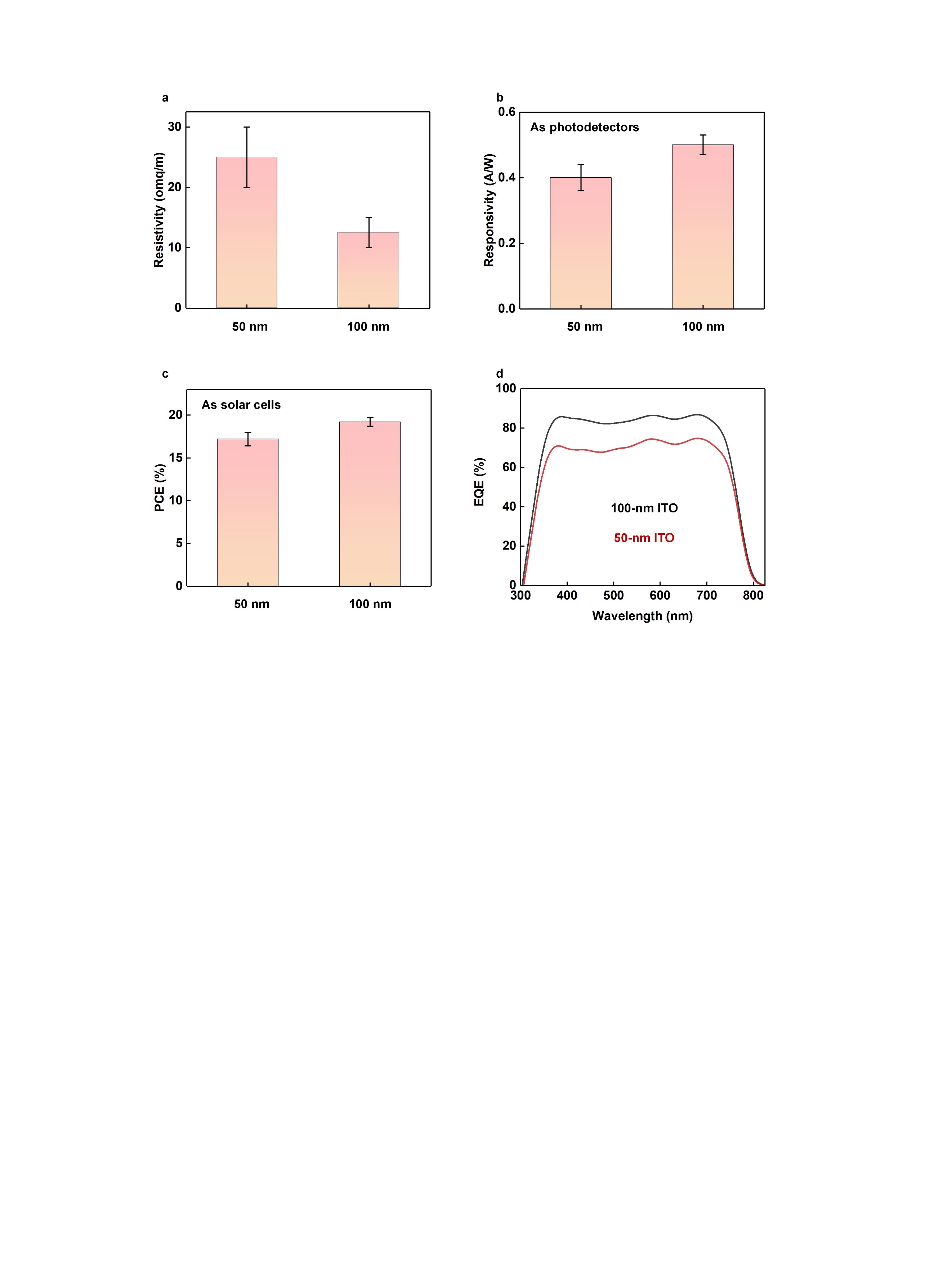 |
| --- |
| **Figure S14. Device performance decay due to the ITO thickness reduction.** (**a**) Statistical resistivity comparison between ITO with different thicknesses. Due to the thickness reduction, 50-nm ITO shows a higher resistivity when compared with that of the 100-nm ITO. Number of experiments, n = 5. (**b**) Responsivity, (**c**) power conversion efficiency (PCE), and (**d**) EQE of perovskite devices with different ITO thicknesses. The responsivity of the photodiode with 50-nm and 100-nm ITO reach 0.4 A W^-1^ and 0.5 A W^-1^, respectively. PCE of the photodiode with 50-nm and 100-nm ITO reach 17% and 19%, respectively. Results reveal an acceptable performance loss due to the limited device area despite the resistivity increment. The responsivity measurements are carried out under -0.5 V bias with a 720-nm incident light. Number of experiments, n = 5 for each type of device. |

To achieve high-selectivity conjugated-BIC resonance and low-loss waveguide mode, we propose to use ITO and SnO_2_ with a thickness of 50 nm and 50 nm, respectively. Conventionally, perovskite optoelectronics would adopt a thicker ITO layer to reduce the charged carrier loss while ensuring sufficient optical transmission[13]. To quantify the optoelectronic losses of our perovskite photodetectors with 50-nm-thick ITO, we systematically study the performance of both types of devices. Figure S14a shows the statistical resistivity of both types of commercial ITO substrates. Results show that the resistivity of the 50-nm ITO is around one-fold higher than that of the 100-nm ITO. This is due to that the resistance of the ITO can be expressed as $R=\frac{\rho L}{S}$, where $R$ is the resistance, $\rho$ is the resistivity, $L$ is the length along the electron propagation, and $S$ is the cross-section area. Since the electrons are planarly propagating in the device for signal output, $S$ is thus the cross-section area of the ITO layer. In this case, $S$ will decrease with the increment of ITO thickness, resulting in the increment of resistance. We then characterize the device performance based on both types of ITO substrates. Note that we adopt the same ITO/SnO_2_/perovskite/Spiro-MeOTAD/Au structure for all devices based on both types of ITO substrates. The thicknesses and deposition conditions for all other layers are controlled to be the same. Meanwhile, this type of device structure can function as both photodetectors and solar cells. To better reveal the impact on the optoelectronic performance by reducing the thickness of ITO, we characterize the same device working as both photodetectors and solar cells regarding their responsivity, PCE, and EQE before the nano-fabrication process. Figure S14b shows the statistical responsivity of both types of devices as photodetectors under the same measurement conditions. Besides, the statistical PCE and EQE of both types of devices as solar cells are also shown in Figure S14c and S14d, respectively. Although a performance drop can be evident, the drop is acceptable considering a limited ITO electrode area is used in this study. Devices with 50-nm ITO still functionally work and give sufficient output performance. By comparing the device performance of both types of devices with different ITO thicknesses, we demonstrate that the reduction of ITO thickness is safe for maintaining the optoelectronic performance of our ultra-narrowband perovskite photodiodes.

**Figure S15**

| 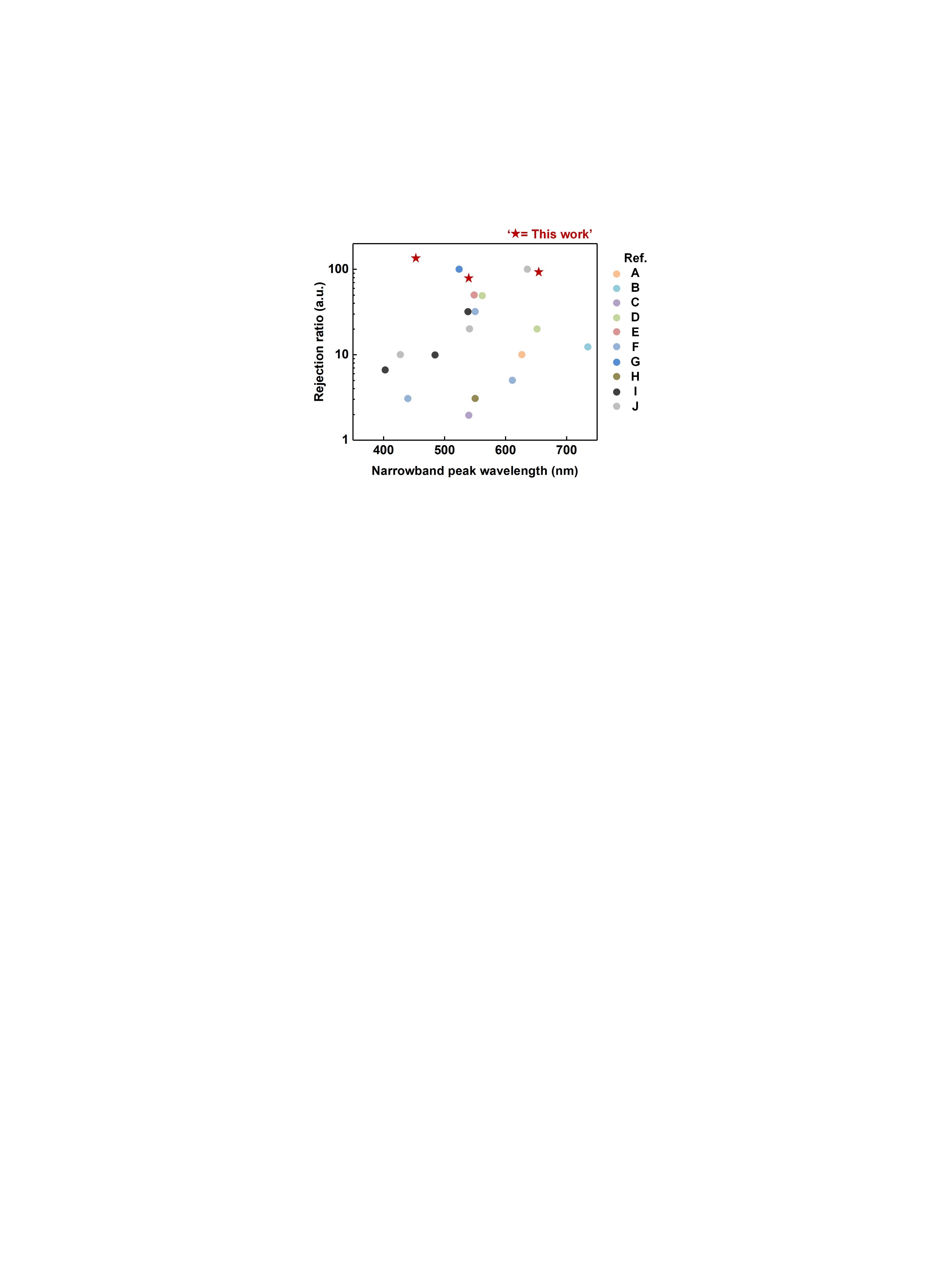 |
| --- |
| **Figure S15. Rejection ratio comparison between our ultra-narrowband perovskite photodiodes with the state-of-art narrowband perovskite photodetectors.** Our ultra-narrowband perovskite photodetectors show a high rejection ratio due to the high selectivity of the conjugated-BIC optical grating. This mechanism is fundamentally different from the conventional mechanisms that enable narrowband photodetection in perovskites. A, ref. [14]; B, ref. [15]; C, ref. [16]; D, ref. [17]; E, ref. [18]; F, ref. [19]; G, ref. [20]; H, ref. [21]; I, ref. [22]; J, ref. [23]. |

An important criterion for evaluating narrowband photodiodes in the spectrometer is how well they can distinguish the light outside of the wavelength of interest. In other words, the narrowband photodiodes should output a minimum photocurrent outside of their narrowband response wavelength range to show an optimized wavelength selectivity, and this can be described as the rejection ratio. The rejection ratio is calculated by $RR=\frac{R_{peak}}{R_{adjcent}}$ where $RR$ is the rejection ratio, $R_{peak}$ is the responsivity peak responsivity, and $R_{adjcent}$ is the responsivity at a particular wavelength outside of the target spectral range[24]. We find that our ultra-narrowband perovskite photodetectors exhibit a high rejection ratio of up to 125. Compared with the photocurrent at the resonant wavelength, this current only takes up ~1% on average, surpassing the previously reported perovskite narrowband photodiodes (Figure S15). We attribute this excellent rejection ratio to the fact that only light at the resonant wavelength can be coupled into the waveguide, while light at other wavelengths will transmit through the grating into the free space. Contrarily, convention perovskite narrowband photodiodes with carrier recombination narrowing mechanism will entirely absorb light with different wavelengths. In this scenario, carriers generated by different-energy photons will partially recombine to realize a narrowband response. Therefore, output current outside of the wavelength-of-interest range cannot be ignored, showing a relatively low rejection ratio.

**Figure S16**

| 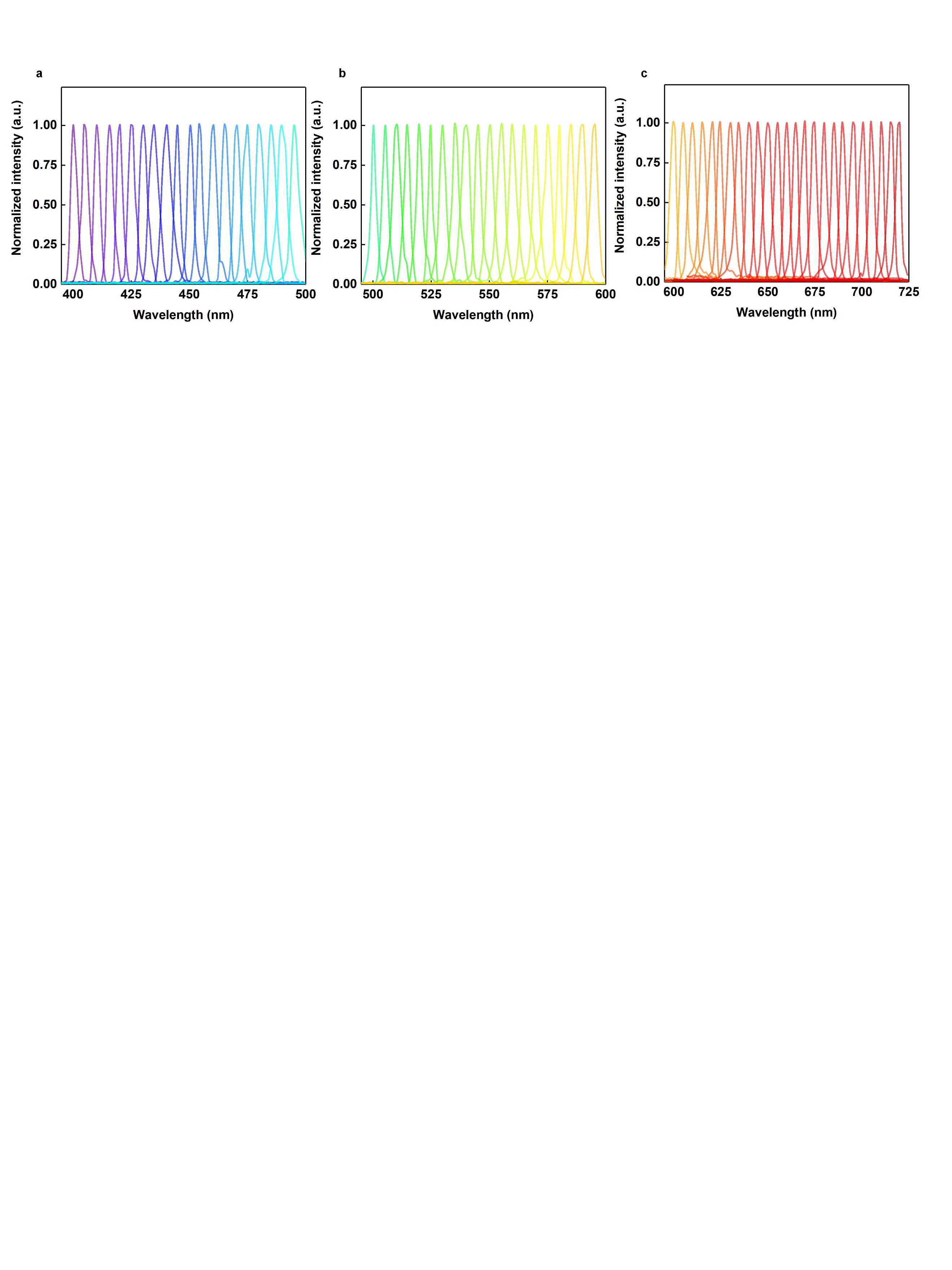 |
| --- |
| **Figure S16. Experimental spectral photocurrent of the ultra-narrowband perovskite photodiodes with different resonant wavelengths.** Experimental Spectral photocurrent of the ultra-narrowband perovskite photodiodes with resonant wavelengths ranging from (**a**) 400 to 500 nm, (**b**) 500 to 600 nm, and (**c**) 600 to 720 nm. |

The experimental spectral resolution of our integrated spectrometer was shown to be around 4 nm based on the full-width at half-maximum (FWHM) of the ultra-narrowband photodiode spectral response (Figure S16). In principle, the resolution of our integrated spectrometer is mainly determined by two factors: the light extraction process and the light propagation process. For the light extraction process, the linewidth of the on-resonant light is highly related to the quality of the conjugated-BIC photonics, including the accuracy of the grating width/period value, the thickness of each layer, the roughness of the grating surface/sidewall, and others. As we have shown, the linewidths of the transmission dips in the calculated transmission spectra of the conjugated-BIC photonics range from 1 to 3 nm (Figure S4), indicating that monochromatic light with FWHM around 1 to 3 nm can be extracted. Through fabrication process optimization, we have experimentally demonstrated conjugated-BIC photonics with transmission dip linewidths range from 2-4 nm (Figure S5). To further improve the spectral resolution by optimizing the light extraction processes, we anticipate that the EBL processes should be further optimized to precisely control the morphology of the conjugated-BIC photonics. Besides, the refractive indices of the materials used in the conjugated-BIC photonics should also be optimized to guarantee sufficient light extraction ability. For the light propagation process, photon scattering in the waveguide can also broaden the extracted monochromatic lights, which can directly deteriorate the spectral resolution of the integrated spectrometers. In this case, it is important to control the morphology of the waveguide to minimize the photon scattering. In this work, we have discussed how the morphology of SnO_2_ layer used in the waveguide affects the spectral resolution of our devices. As we have shown in Figure S8, the spectral resolution of the ultra-narrowband device based on SnO_2_ nanoparticles (NPs) was characterized to be 30.2 nm due to the severe photon scattering by the SnO_2_ NPs. As comparison, device based on sol-gel SnO_2_ film showed a smoother morphology and the spectral resolution was largely improved to 3.6 nm. To further improve the spectral resolution by optimizing the light propagation processes, we anticipate that the materials that used in the waveguide should be replaced to minimize the optical attenuation.

**Figure S17**

| 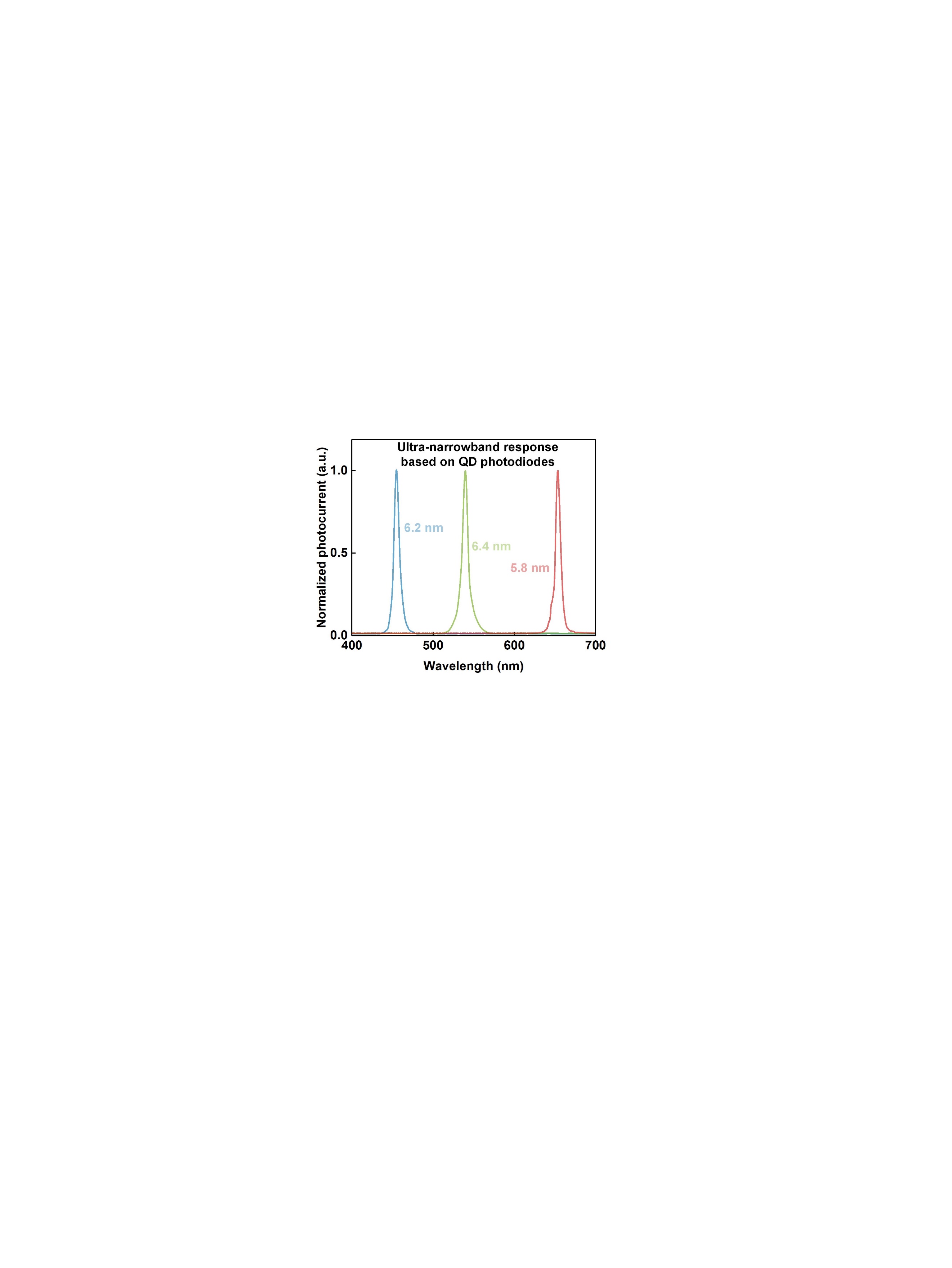 |
| --- |
| **Figure S17. Normalized spectral photocurrent of the ultra-narrowband QD photodiodes with three different resonant wavelengths.** Results show that the ultra-narrowband photodiodes based on QDs can also realize monochromatic light detection, proving that the proposed platform possesses semiconductor substitutability. |

**Figure S18**

| 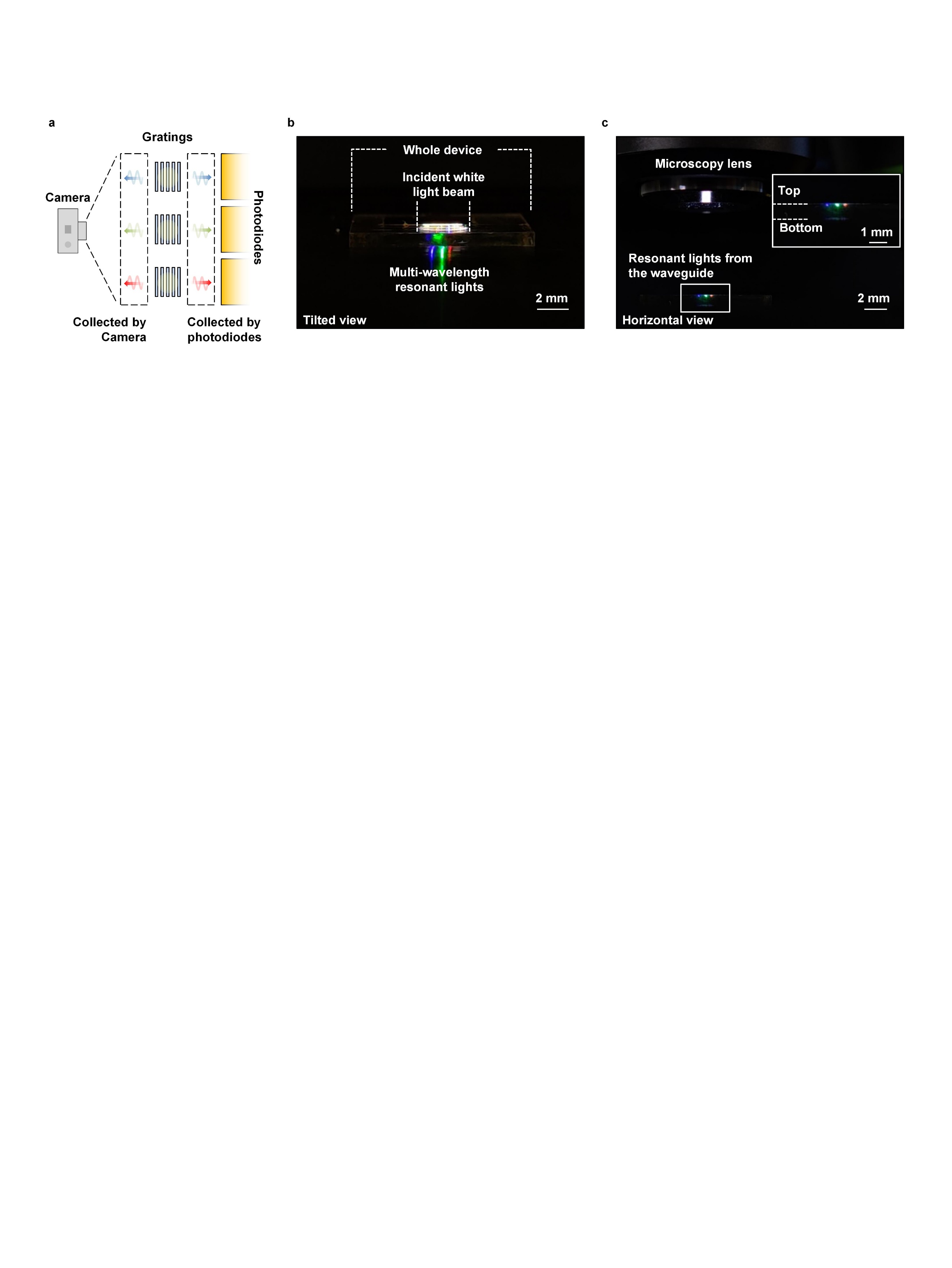 |
| --- |
| **Figure S18. Ultra-narrowband perovskite photodiode arrays integrated three resonant wavelengths.** (**a**) Schematic of the ultra-narrowband perovskite photodiode array measurement. The measurement is carried out based on ultra-narrowband perovskite photodiode arrays integrated with three resonant wavelengths. Three monochromatic lights with different resonant wavelengths from the white light that is projected onto the device can be extracted. The monochromatic lights will propagate in two opposite directions with one of them being collected by the integrated photodiode arrays while the other being collected by the camera. (**b**) An optical image from the tilted view. A beam of white light is projected onto the device with three different resonant-wavelength optical gratings, resulting in the coupling of corresponding resonant lights into the waveguide. The resonant lights then propagate parallelly (in the in-plane direction) into opposite directions. The resonant lights are absorbed by the perovskite layer for photocurrent in one direction while leaving the device and emitting into the free space in the other direction. This result shows the multi-wavelength integration ability of our ultra-narrowband perovskite photodetectors. (**c**) An optical image from the horizontal view. The resonant lights are emitting from the upper layer of the device, indicating that the resonant lights are confined in the waveguide rather than leaking to the substrate during the propagation in the device. Inset, the zoom-in horizontal image of the ultra-narrowband photodetector. |

One of the major drawbacks of conventional perovskite narrowband photodetectors is that each detection wavelength requires a unique combination of perovskite absorber thickness and composition[14, 23, 25]. Therefore, the integration of multi-wavelength perovskite narrowband photodetectors on one chip can be extremely difficult, severely impeding their use in advanced applications like spectrometers. Similarly, narrowband perovskite photodetectors using a self-filtered mechanism with additional filtering perovskite layers or photodetectors also face the same problem of on-chip integration[26].

Compared with the conventional perovskite narrowband photodetectors, our ultra-narrowband perovskite photodiodes can be facilely integrated for applications like spectrometers. Specifically, the broadband perovskite photodiode arrays adopt the same device configuration, and can be fabricated at once. Through EBL, conjugated-BIC gratings with different resonant wavelengths can be integrated with the corresponding broadband photodiodes to form ultra-narrowband photodiode arrays. Figure S18a shows the structure of the ultra-narrowband perovskite photodiode arrays integrated with three resonant wavelengths. In the tilted-view optical image (Figure S18b), the photodetector is placed underneath the optical microscope to locate the position of the BIC gratings. A beam of white light generated by the microscope is projected onto the three BIC gratings simultaneously, where wavelength-designed resonant lights in the white light can be coupled into the waveguide through the BIC-induced waveguide-grating resonance. We integrate three devices with different narrowband response ranges using the same perovskite photodetector configuration in this device. Observing from the opposite side of the perovskite photodetectors, we can clearly see three beams of light with different wavelengths due to the opposite propagation direction. Meanwhile, no light can be observed from the side with the perovskite photodetectors, resulting from the absorption of the perovskite layer with a much larger n. From the horizontal-view optical image (Figure S18c), it clearly shows that the resonant lights emit from the top layer at the edge of the whole device. This indicates that the light can be confined in the waveguide, which is located at the surface layer of the substrate, rather than leaking into the substrate. This result sufficiently proves the on-chip integration capability of our perovskite photodetectors for multi-wavelength narrowband photodetection. Meanwhile, incident light no longer requires projection through the transparent glass substrate, providing extra flexibility to the design of perovskite device configuration during the on-chip integration.

**Figure S19**

| 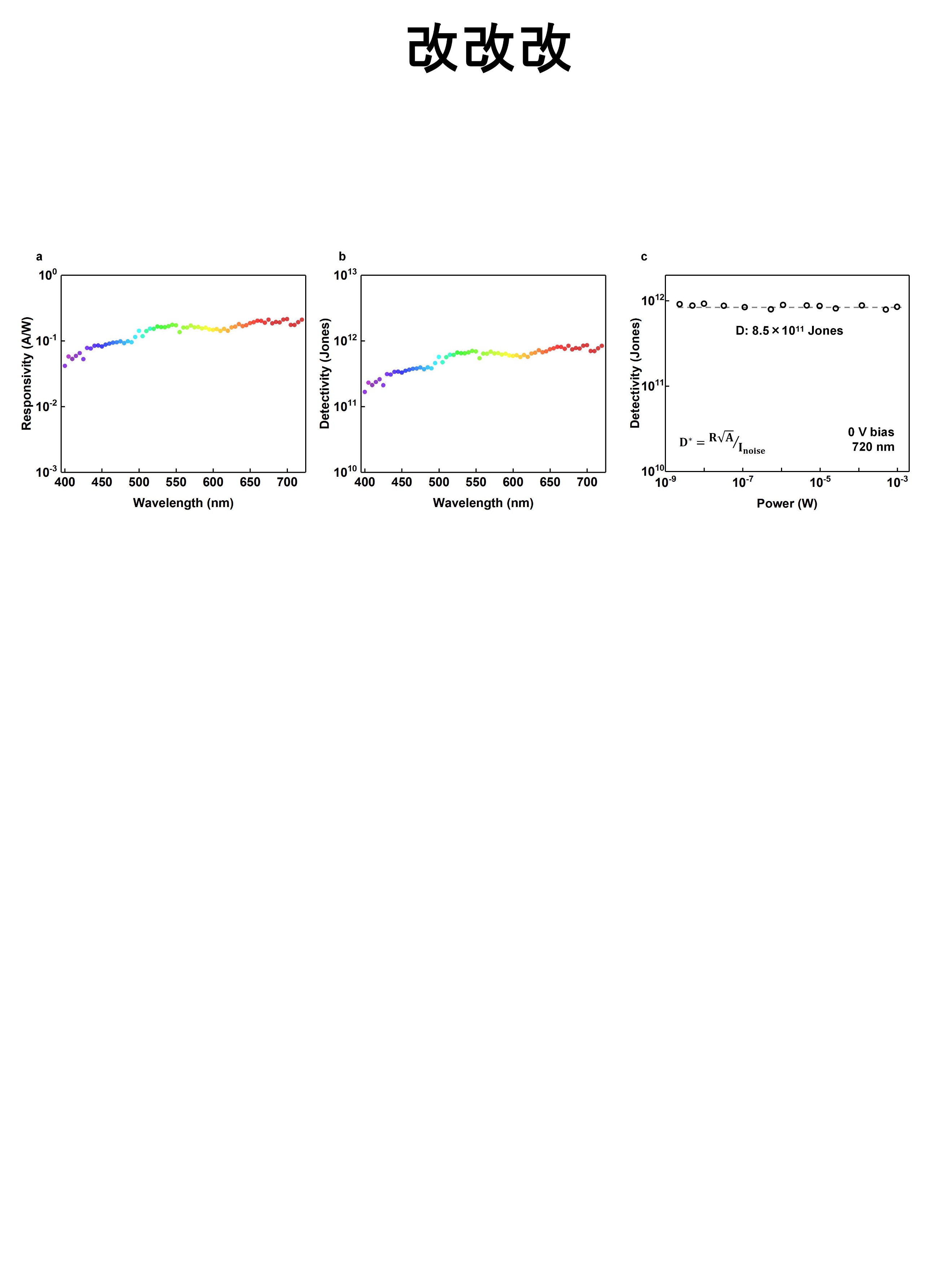 |
| --- |
| **Figure S19. Characterizations of the ultra-narrowband perovskite photodiodes.** (a) Wavelength-dependent responsivity, (b) wavelength-dependent detectivity, and (c) the detectivity under 720 nm of the ultra-narrowband perovskite photodiodes. Responsivity and detectivity are also characterized to be as high as 0.2 A W^-1^ and 8.5×10^11^ Jones. |

**Table S1. Structure of the conjugated-BIC photonics with different on-resonant wavelengths and the optical loss of the waveguide at the corresponding wavelengths**

| **On-resonant wavelength (nm)** | **Period (nm)** | **Width (nm)** | **Loss (dB cm^-1^)** |
| --- | --- | --- | --- |
| 400 | 248 | 110 | 332.9 |
| 405 | 252 | 110 | 329.8 |
| 410 | 256 | 120 | 326.8 |
| 415 | 260 | 120 | 323.8 |
| 420 | 264 | 120 | 320.8 |
| 425 | 268 | 120 | 317.9 |
| 430 | 272 | 130 | 315.1 |
| 435 | 276 | 130 | 312.0 |
| 440 | 280 | 130 | 308.8 |
| 445 | 284 | 130 | 305.6 |
| 450 | 287 | 140 | 368.6 |
| 455 | 291 | 140 | 361.2 |
| 460 | 295 | 140 | 354.5 |
| 465 | 299 | 140 | 348.5 |
| 470 | 303 | 140 | 343.1 |
| 475 | 306 | 140 | 338.3 |
| 480 | 310 | 150 | 334.0 |
| 485 | 314 | 150 | 330.1 |
| 490 | 317 | 150 | 326.6 |
| 495 | 321 | 150 | 323.5 |
| 500 | 325 | 150 | 320.6 |
| 505 | 328 | 150 | 318.1 |
| 510 | 332 | 150 | 315.7 |
| 515 | 336 | 150 | 313.6 |
| 520 | 339 | 150 | 311.8 |
| 525 | 343 | 140 | 310.0 |
| 530 | 346 | 140 | 308.5 |
| 535 | 350 | 140 | 307.1 |
| 540 | 354 | 140 | 305.8 |
| 545 | 357 | 160 | 304.7 |
| 550 | 361 | 230 | 303.6 |
| 555 | 364 | 250 | 302.7 |
| 560 | 368 | 260 | 301.8 |
| 565 | 371 | 270 | 301.1 |
| 570 | 374 | 280 | 300.4 |
| 575 | 376 | 290 | 297.9 |
| 580 | 378 | 290 | 292.4 |
| 585 | 382 | 300 | 287.5 |
| 590 | 386 | 310 | 283.2 |
| 595 | 390 | 320 | 279.3 |
| 600 | 394 | 100 | 276.0 |
| 605 | 398 | 150 | 273.0 |
| 611 | 401 | 180 | 270.5 |
| 615 | 404 | 90 | 268.3 |
| 620 | 408 | 120 | 266.5 |
| 625 | 411 | 120 | 265.0 |
| 630 | 414 | 260 | 263.9 |
| 635 | 417 | 250 | 263.2 |
| 640 | 421 | 230 | 265.2 |
| 645 | 424 | 260 | 262.2 |
| 650 | 427 | 280 | 261.7 |
| 654 | 430 | 290 | 261.6 |
| 660 | 434 | 310 | 261.8 |
| 665 | 437 | 340 | 262.2 |
| 670 | 440 | 340 | 262.7 |
| 675 | 444 | 330 | 263.5 |
| 680 | 447 | 370 | 264.4 |
| 685 | 450 | 360 | 265.4 |
| 690 | 454 | 370 | 266.6 |
| 695 | 457 | 380 | 267.9 |
| 700 | 460 | 380 | 269.3 |
| 705 | 463 | 380 | 270.8 |
| 710 | 467 | 370 | 272.5 |
| 715 | 470 | 370 | 274.2 |
| 720 | 473 | 380 | 276.0 |

**Table S2. Overall performance comparison of the reported perovskite narrowband photodetectors.**

| **Device configuration** | **Detector type** | **Mechanism** | **Center wavelength** | **FWHM** | **R** | **Response speed** | **Reference** |
| --- | --- | --- | --- | --- | --- | --- | --- |
| **conjugated-BIC-waveguide-**  **ITO/ETL/PVK/HTL/Au** | **Diodes** | **Conjugated-BIC** | **400-720 nm tunable** | **˂4 nm** | **0.2** A W^-1^  **(-0.5 V)** | **0.1 µs**  **(0.01 cm^2^)** | **This work** |
| FTO/HTL/PVK/ ETL/Ag | Diodes | CCN | 800 nm  (MAPbI_3_)  715 nm  (MAPbI_2_Br)  615 nm  (MAPbIBr_2_) | >30 nm | 0.0637 A W^-1^ | 6.9 µs | Ref. [14] |
| ITO/HTL/PVK/ETL/Ag | Diodes | CCN | 800 nm | >50 nm | 0.076 A W^-1^  (-1V) | 1.167 µs | Ref. [27] |
| ITO/HTL/PVK/ETL/Ag | Diodes | CCN | 450 nm  (MAPbI_1.4_Br_0.6_)  550 nm  (MAPbIBr_2_)  650 nm  (MAPbI_2_Br) | 100 nm | 0.05 A W^-1^  (-0.5 V) | 1.5 µs | Ref. [28] |
| PVK/Glass/ITO/HTL/PVK/ETL/Cu | Diodes | Self-filter | 750 nm | 28 nm | 0.076 A W^-1^ | 0.6 µs  (0.08 cm^2^) | Ref. [26] |
| PVK/Glass/ITO/HTL/PVK/ETL/Ag | Diodes | Self-filter | 550 nm  (MAPbBr_2.4_I_0.6_)  625 nm  (MAPbBr_1.5_I_1.5_)  750 nm  (MAPbI_3_) | 44 nm | 0.33 A W^-1^  (-1.5 V) | 180 µs | Ref. [15] |
| NPB-LiF layer/Glass/ITO/HTL/PVK/ETL/Ag | Diodes | Microcavi-ty resonance | 800 nm  (CsPb_0.5_Sn_0.5_I_3_) | 50 nm | 0.27 A W^-1^ | 0.65 µs | Ref. [29] |
| Ga/PVK/Au | Conductor | CCN | 425 nm  (MAPbCl_3_)  530 nm  (MAPbBr_1.5_Cl_1.5_)  635 nm  (MAPbI_2.57_Br_0.43_) | 20 nm | N/A | >20 µs | Ref. [23] |
| Au/PVK/Au | Conductor | CCN | 550 nm | >24 nm | 1600 A/W  (-5 V) | 130000 µs | Ref. [18] |
| Au/PVK/Au | Conductor | CCN | 440 nm  (Cs_2_SnBr_1.14_Cl_4.86_)  530 nm  (Cs_2_SnBr_3.85_Cl_2.15_)  600 nm  (Cs_2_SnBr_6_) | 45 nm | N/A | 4300 µs | Ref. [30] |
| ITO/PVK/Au | Conductor | CCN | 540 nm | 20 nm | 0.9 A W^-1^ | 170000 µs | Ref. [16] |
| Au/PVK/Au | Conductor | CCN | 560 nm | 20 nm | 0.56 A W^-1^  (-5 V) | 166000 µs | Ref. [31] |
| ITO/PVK/Au | Conductor | CCN | 425 nm  (BA_2_PbBr_4_)  560 nm  (BA_2_PbI_4_)  700 nm  (BA_2_MA_4_Pb_5_I_16_) | 20 nm | N/A | 74000 µs | Ref. [17] |
| Au/PVK/Au | Conductor | CCN | 400 nm  (CsPbCl_3_)  500 nm  (CsPbBr_2_Cl)  600 nm  (CsPbBr_2_I) | >12 nm | 0.05 A W^-1^  (5 V) | >4000 µs | Ref. [19] |
| Au/PVK/Au | Conductor | CCN | 550 nm | >13.6 nm | 0.055 A W^-1^  (9 V) | 26000 µs | Ref. [20] |
| FTO/PVK/Au | Conductor | CCN | 560 nm | 50 nm | N/A | >3 µs | Ref. [21] |
| ITO/PVK/ITO | Conductor | CCN | 410 nm  (MAPbCl_3_)  550 nm  (MAPbBr_3_)  830 nm  (MAPbI_3_) | 33 nm | 1640 A W^-1^ | 20 µs | Ref. [22] |

PVK, perovskite. ETL, electron transporting layer. HTL, hole transporting layer. FWHM, full-width at half maximum. R, responsivity.

We systematically study the overall performance of the perovskite narrowband photodetectors in the field. From the comparison, we show that the novel narrowband photodetection we proposed exhibits several merits, including the capacity for multi-wavelength on-chip integration, tunable narrowband photodetection wavelengths, ultra-narrowband photo-response, and fast speed. The responsivity is on par with those of the reported perovskite narrowband photodiodes.

**Reference**

1. Xiong, L., et al., *Review on the Application of SnO2 in Perovskite Solar Cells.* Advanced Functional Materials, 2018. **0**(0): p. 1802757.

2. Lei, Y., et al., *A fabrication process for flexible single-crystal perovskite devices.* Nature, 2020. **583**(7818): p. 790-795.

3. Chen, S., et al., *Atomic scale insights into structure instability and decomposition pathway of methylammonium lead iodide perovskite.* Nature Communications, 2018. **9**(1): p. 4807.

4. Li, S., et al., *Realizing CsPbBr3 Light-Emitting Diode Arrays Based on PDMS Template Confined Solution Growth of Single-Crystalline Perovskite.* The Journal of Physical Chemistry Letters, 2020. **11**(19): p. 8275-8282.

5. Fan, Y., et al., *Enhanced Multiphoton Processes in Perovskite Metasurfaces.* Nano Letters, 2021. **21**(17): p. 7191-7197.

6. Wang, K., et al., *Micro- and Nanostructured Lead Halide Perovskites: From Materials to Integrations and Devices.* Advanced Materials, 2020. **n/a**(n/a): p. 2000306.

7. Wang, Y., et al., *Highly Controllable Etchless Perovskite Microlasers Based on Bound States in the Continuum.* ACS Nano, 2021. **15**(4): p. 7386-7391.

8. Ollearo, R., et al., *Ultralow dark current in near-infrared perovskite photodiodes by reducing charge injection and interfacial charge generation.* Nature Communications, 2021. **12**(1): p. 7277.

9. Bao, C., et al., *Bidirectional optical signal transmission between two identical devices using perovskite diodes.* Nature Electronics, 2020. **3**(3): p. 156-164.

10. Shen, L., et al., *A Self-Powered, Sub-nanosecond-Response Solution-Processed Hybrid Perovskite Photodetector for Time-Resolved Photoluminescence-Lifetime Detection.* Advanced Materials, 2016. **28**(48): p. 10794-10800.

11. Liu, H., et al., *Double-Side Crystallization Tuning to Achieve over 1 µm Thick and Well-Aligned Block-Like Narrow-Bandgap Perovskites for High-Efficiency Near-Infrared Photodetectors.* Advanced Functional Materials, 2021. **n/a**(n/a): p. 2010532.

12. Xu, X., et al., *High-Performance Near-IR Photodetector Using Low-Bandgap MA0.5FA0.5Pb0.5Sn0.5I3 Perovskite.* Advanced Functional Materials, 2017. **27**(28): p. 1701053.

13. Jiang, Q., et al., *Surface passivation of perovskite film for efficient solar cells.* Nature Photonics, 2019. **13**(7): p. 460-466.

14. Wang, J., et al., *Self-Driven Perovskite Narrowband Photodetectors with Tunable Spectral Responses.* Advanced Materials, 2021. **33**(3): p. 2005557.

15. Qiao, S., et al., *High-Responsivity, Fast, and Self-Powered Narrowband Perovskite Heterojunction Photodetectors with a Tunable Response Range in the Visible and Near-Infrared Region.* ACS Applied Materials & Interfaces, 2021. **13**(29): p. 34625-34636.

16. Wang, J., et al., *Controllable Growth of Centimeter-Sized 2D Perovskite Heterostructures for Highly Narrow Dual-Band Photodetectors.* ACS Nano, 2019.

17. Li, J., et al., *Self-trapped state enabled filterless narrowband photodetections in 2D layered perovskite single crystals.* Nature Communications, 2019. **10**(1): p. 806.

18. Wang, H., et al., *2D perovskite narrowband photodetector arrays.* Journal of Materials Chemistry C, 2021. **9**(34): p. 11085-11090.

19. Xue, J., et al., *Narrowband Perovskite Photodetector-Based Image Array for Potential Application in Artificial Vision.* Nano Letters, 2018. **18**(12): p. 7628-7634.

20. Wu, Y., et al., *Perovskite photodetectors with both visible-infrared dual-mode response and super-narrowband characteristics towards photo-communication encryption application.* Nanoscale, 2018. **10**(1): p. 359-365.

21. Rao, H.-S., et al., *In Situ Growth of 120 cm2 CH3NH3PbBr3 Perovskite Crystal Film on FTO Glass for Narrowband-Photodetectors.* Advanced Materials, 2017. **29**(16): p. 1602639.

22. Saidaminov, M.I., et al., *Perovskite Photodetectors Operating in Both Narrowband and Broadband Regimes.* Advanced Materials, 2016. **28**(37): p. 8144-8149.

23. Fang, Y., et al., *Highly narrowband perovskite single-crystal photodetectors enabled by surface-charge recombination.* Nature Photonics, 2015. **9**(10): p. 679.

24. Pecunia, V., *Efficiency and spectral performance of narrowband organic and perovskite photodetectors: a cross-sectional review.* Journal of Physics: Materials, 2019. **2**(4): p. 042001.

25. Li, L., et al., *Recent Advances in Perovskite Photodetectors for Image Sensing.* Small, 2021. **17**(18): p. 2005606.

26. Li, L., et al., *Self-Filtered Narrowband Perovskite Photodetectors with Ultrafast and Tuned Spectral Response.* Advanced Optical Materials, 2017. **5**(22): p. 1700672.

27. Lan, Z., et al., *Narrowband Near-Infrared Perovskite/Polymer Hybrid Photodetectors.* ACS Applied Materials & Interfaces, 2020.

28. Lin, Q., et al., *Filterless narrowband visible photodetectors.* Nature Photonics, 2015. **9**(10): p. 687-694.

29. Cao, F., et al., *Bionic Detectors Based on Low-Bandgap Inorganic Perovskite for Selective NIR-I Photon Detection and Imaging.* Advanced Materials, 2020. **32**(6): p. 1905362.

30. Zhou, J., et al., *Lead-Free Perovskite Derivative Cs2SnCl6−xBrx Single Crystals for Narrowband Photodetectors.* Advanced Optical Materials, 2019. **7**(10): p. 1900139.

31. Li, L., et al., *Filterless Polarization-Sensitive 2D Perovskite Narrowband Photodetectors.* Advanced Optical Materials, 2019. **7**(23): p. 1900988.
